# Genome mining and molecular characterization of the biosynthetic gene cluster of a diterpenic meroterpenoid, 15-deoxyoxalicine B, in *Penicillium canescens*†
†Electronic supplementary information (ESI) available. See DOI: 10.1039/c5sc01965f


**DOI:** 10.1039/c5sc01965f

**Published:** 2015-08-06

**Authors:** Junko Yaegashi, Jillian Romsdahl, Yi-Ming Chiang, Clay C. C. Wang

**Affiliations:** a Department of Pharmacology and Pharmaceutical Sciences , School of Pharmacy , University of Southern California , Los Angeles , California 90089 , USA . Email: clayw@usc.edu; b Graduate Institute of Pharmaceutical Science , Chia Nan University of Pharmacy and Science , Tainan 71710 , Taiwan; c Department of Chemistry , College of Letters, Arts and Sciences , University of Southern California , Los Angeles , California 90089 , USA

## Abstract

Meroterpenoids are a class of secondary metabolites that are produced from polyketide and terpenoid precursors. 15-Deoxyoxalicine B (**1**) belongs to one structural group consisting of a unique pyridinyl-α-pyrone polyketide subunit and a diterpenoid subunit connected through a characteristic asymmetric spiro carbon atom. An understanding of the genes involved in the biosynthesis of this class of compounds should provide a means to facilitate engineering of second-generation molecules and increasing production of first-generation compounds. We found that the filamentous fungus *Penicillium canescens* produces 15-deoxyoxalicine B (**1**). Using targeted gene deletions, we have identified a cluster of 12 responsible contiguous genes. This gene cluster includes one polyketide synthase gene which we have designated *olcA*. Chemical analysis of wild-type and gene deletion mutant extracts enabled us to isolate and characterize 7 additional metabolites that are either intermediates or shunt products of the biosynthetic pathway. Two of the compounds identified have not been reported previously. Our data have allowed us to propose a biosynthetic pathway for 15-deoxyoxalicine B (**1**).

## Abstract 2

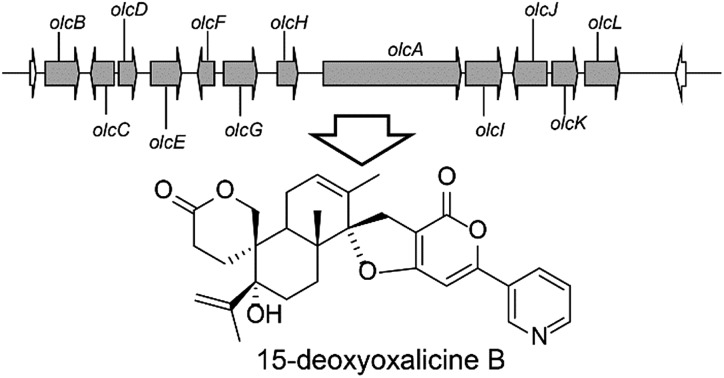
The biosynthetic pathway of a diterpenic meroterpenoid was elucidated by combination of genome mining, gene targeting, and natural product chemistry.

## Introduction

Natural products have served as important sources of bioactive molecules for decades.^1^ Most natural products are secondary metabolites (SMs) of microorganisms and plants, and their wide range of bioactivities are derived from their tremendous structural diversity. Intriguingly, the core structures of these natural products are assembled from simple precursors, and structural diversity is the result of reactions catalyzed by different enzymes that are responsible for numerous modifications in the course of the biosynthesis process. Classes of compounds that illustrate such structural diversity include terpenoids and polyketides, which are built and decorated upon core structures assembled by terpene cyclases and polyketide synthases (PKS), respectively.^2^,^3^ Moreover, these two classes of enzymes can work together in sequence to produce hybrid molecule meroterpenoids. Meroterpenoids are found widely in plants, fungi, and bacteria and show a broad range of bioactivities.^4^ Examples of known meroterpenoids with bioactivities that can be applied for therapeutic uses include the acetylcholinesterase inhibitor territrem B for the potential treatment of Alzheimer's disease,^5^,^6^ the acyl-CoA cholesterol acetyltransferase inhibitor pyripyropene A for the potential treatment and prevention of atherosclerosis,^7^,^8^ and mycophenolic acid as a clinically used immunosuppressive agent.^9^–^11^

**Fig. 1:**
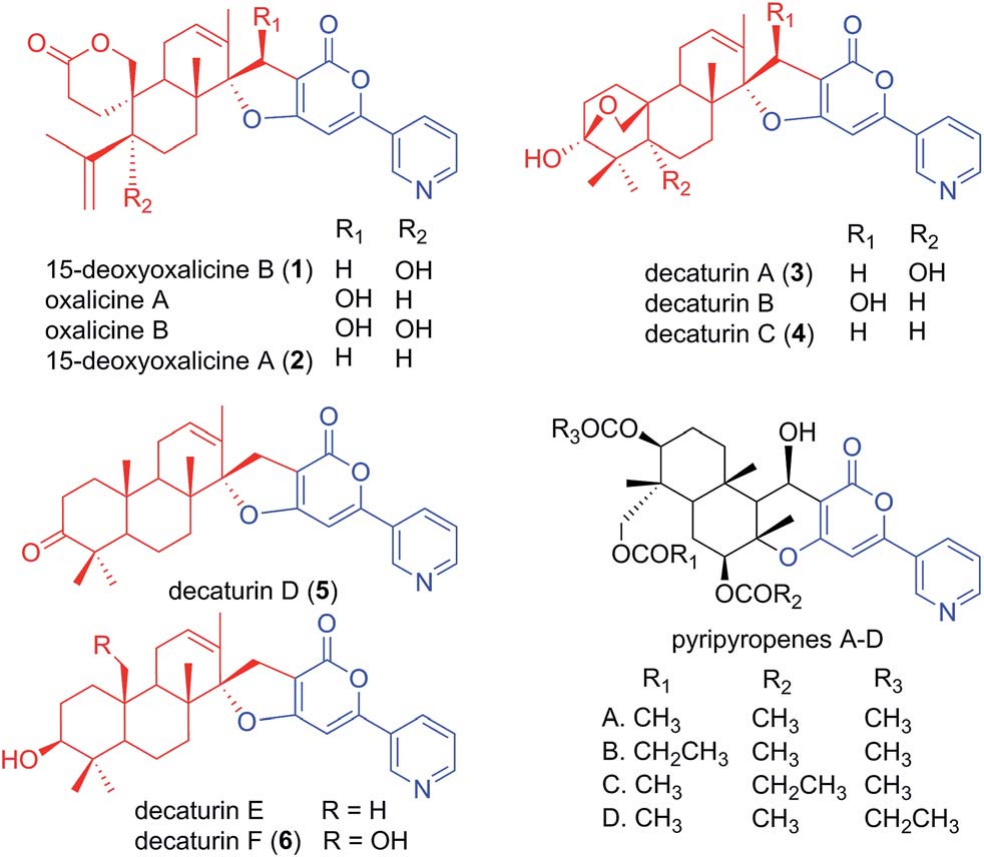
Structurally related fungal meroterpenoids. The polyketide portion is shown in blue, and the diterpenic terpenoid portion in red. Compounds **1–6** were isolated in this study.

**Scheme 1:**
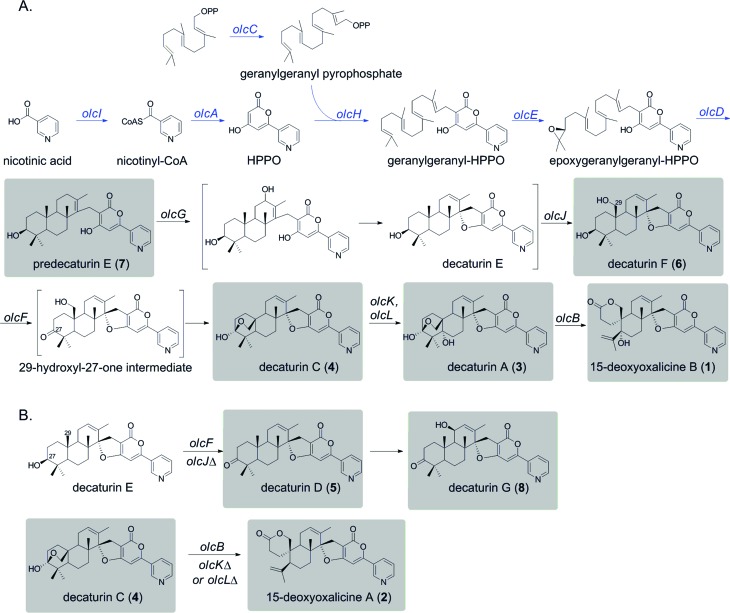
Proposed biosynthetic pathways for **1** and related shunt products. All natural products isolated from this study are indicated by shaded boxes. (A) Proposed biosynthetic pathway leading to the production of **1**. Parts of the pathway deduced based on similarity to the pyripyropene A biosynthetic pathway are indicated with blue arrows. Brackets indicate hypothetical parts of the pathway. (B) Proposed shunt pathways from decaturin E in *olcJ*Δ strain and decaturin C (**4**) in *olcK*Δ or *olcL*Δ strains.

Although many meroterpenoids have been isolated and characterized, the genes involved in their biosynthesis have been revealed in only a few instances in fungi,^12^–^17^ while the majority of them have yet to be elucidated. Examples of such compounds are oxalicines A and B. They were first isolated from *Penicillium oxalicum* by Ubillas *et al.*^18^,^19^ and were the first of a rare skeletal class of diterpenic meroterpenoids (Fig. 1). Since then, other related compounds such as 15-deoxyoxalicines A, B and decaturins A–F have been isolated from *P. decaturense*,^20^*P. thiersii*,^21^ and/or *P. oxalicum*,^22^ and many of them were shown to have antiinsectan activity against the fall armyworm (*Spodoptera frugiperda*). These compounds form a structurally unique class of natural products, because their basic structure is composed of the following two subunits: a pyridinyl-α-pyrone polyketide subunit (Fig. 1, blue), and a diterpenoid subunit (Fig. 1, red). This pyridinyl-α-pyrone polyketide subunit itself is rare among natural products, having only been found in anibine, a plant metabolite,^23^ and pyripyropenes, a group of potent acyl-CoA cholesterol acyltransferase inhibitors isolated from *Aspergillus fumigatus*.^12^ The early steps of pyripyropene A biosynthesis has been determined previously,^12^ and the formation of the pyridinyl-α-pyrone polyketide subunit is most likely highly similar to that of oxalicines and decaturins. These steps involve the CoA ligase Pyr1 and PKS Pyr2 for the construction of 4-hydroxy-6-(3-pyridinyl)-2*H*-pyran-2-one (HPPO, Scheme 1), followed by the prenyltransferase Pyr6, FAD-dependent monooxygenase Pyr5, and terpene cyclase Pyr4 for the production of deacetyl-pyripyropene E, a precursor of pyripyropene A.^12^ However, instead of the incorporation of a farnesyl group in pyripyropene A biosynthesis, oxalicine and decaturin biosynthesis incorporate a geranylgeranyl group to produce the diterpenoid PKS hybrid. Moreover, the two subunits of the oxalicines and decaturins are connected through a unique and characteristic asymmetric spiro carbon atom, a feature that is lacking in pyripyropenes.

In recent years, the Joint Genome Institute (JGI) has released a number of complete genome sequences for various *Penicillium* species, making it feasible to perform *in silico* analysis of potential SM gene clusters. Genome sequence data analysis shows that this species harbors many core SM biosynthesis genes, suggesting that it has the potential to produce far more SMs than is currently known. Taking advantage of the fact that early steps of pyripyropene A biosynthesis have been determined, we attempted to identify the biosynthetic genes for meroterpenoids that are structurally related to pyripyropene A in *Penicillium* species by genome mining. We found that *P. canescens* was the only *Penicillium* species genome-sequenced by JGI that harbored a complete set of genes homologous to genes in the pyripyropene A biosynthetic gene cluster, suggesting its potential to produce pyripyropene-like meroterpenoids. Interestingly, these genes were surrounded by genes encoding additional potential tailoring enzymes, suggesting that the final product of this biosynthetic gene cluster would be different from pyripyropene A. We grew *P. canescens* on various media and found that cultivation on Czapek's media induced the production of a compound that we isolated and identified as 15-deoxyoxalicine B (**1**). We then developed an efficient gene targeting system for *P*. *canescens*, and this allowed us to identify and characterize a gene cluster containing 12 contiguous genes that are involved in the biosynthesis of 15-deoxyoxalicine B (**1**). Several of the gene deletant strains accumulated chemically stable intermediates or shunt products in sufficient amounts for full structural characterization by spectroscopic methods. These strains were cultivated in large-scale, and we were able to isolate a total of 7 related compounds (**2–8**). Two of these compounds (**7** and **8**, Scheme 1) have not been reported previously. Combined with further bioinformatics analysis, we have proposed a biosynthetic pathway for 15-deoxyoxalicine B.

## Results

### Analysis of the potential gene cluster for the production of a pyripyropene-like meroterpenoid *in silico*

Bioinformatics analysis of *Penicillium* species using the JGI database (; http://genome.jgi-psf.org/programs/fungi/index.jsf) was performed to find potential gene clusters that would be involved in the biosynthesis of pyripyropene-like meroterpenoids. BLAST analysis was performed using as reference the genes that encode the first three responsible enzymes of pyripyropene A biosynthesis: CoA-ligase Pyr1, PKS Pyr2, and prenyltransferase Pyr6. Interestingly, *P. canescens* was the only *Penicillium* species genome-sequenced by JGI that harbored all three genes in close enough proximity in the genome to form a potential gene cluster. Further analysis revealed that the PKS of *P. canescens* which showed high sequence homology to Pyr2, was surrounded by a cluster of genes that contained homologs of genes that encode all the enzymes found in the biosynthetic pathway of pyripyropene A (Table 1). The homologs included Pyr1 (protein ID 437327), Pyr2 (protein ID 400488), Pyr4 (protein ID 437321), Pyr5 (protein ID351329), and Pyr6 (protein ID 410812). These homologs have high sequence identity (≥41%) and similarity (≥57%), highlighting the potential of this species to produce pyripyropene-like meroterpenoids, although there is no report that such compounds have been isolated from *P*. *canescens*.

**Table 1:** Putative function of genes within the 15-deoxyoxalicine B gene cluster and their homologs in *A. fumigatus*

Gene designation	Protein ID^*a*^	*A. fumigatus* homologs (Afu6gxxxxx)	Similarity/identity (%)	Putative function
	410805^*b*^			Cytoskeletal protein adducin
*olcB*	333321			Cytochrome P450 CYP3/CYP5/CYP6/CYP9 subfamilies
*olcC*	351326			Geranylgeranyl pyrophosphate synthase
*olcD*	437321	13950 (*pyr4*)	57/41	Integral membrane protein (terpene cyclase)
*olcE*	351329	13970 (*pyr5*)	74/60	FAD-dependent monooxygenase
*olcF*	367480			Short chain dehydrogenase
*olcG*	393266			Cytochrome P450 CYP3/CYP5/CYP6/CYP9 subfamilies
*olcH*	410812	13980 (*pyr6*)	68/52	Prenyltransferase
*olcA*	400488	13930 (*pyr2*)	59/42	PKS
*olcI*	437327	13920 (*pyr1*)	71/58	CoA ligase
*olcJ*	333335			Cytochrome P450 CYP3/CYP5/CYP6/CYP9 subfamilies
*olcK*	367485			Hydroxylase
*olcL*	351342			Predicted transporter (major facilitator superfamily)
	367486^*b*^			Hypothetical protein

^*a*^Protein IDs as designated in JGI database.

^*b*^These genes are predicted to be outside the gene cluster.

### Detection of pyripyropene-like meroterpenoids in *P. canescens* cultivated on Czapek's medium

**Fig. 2:**
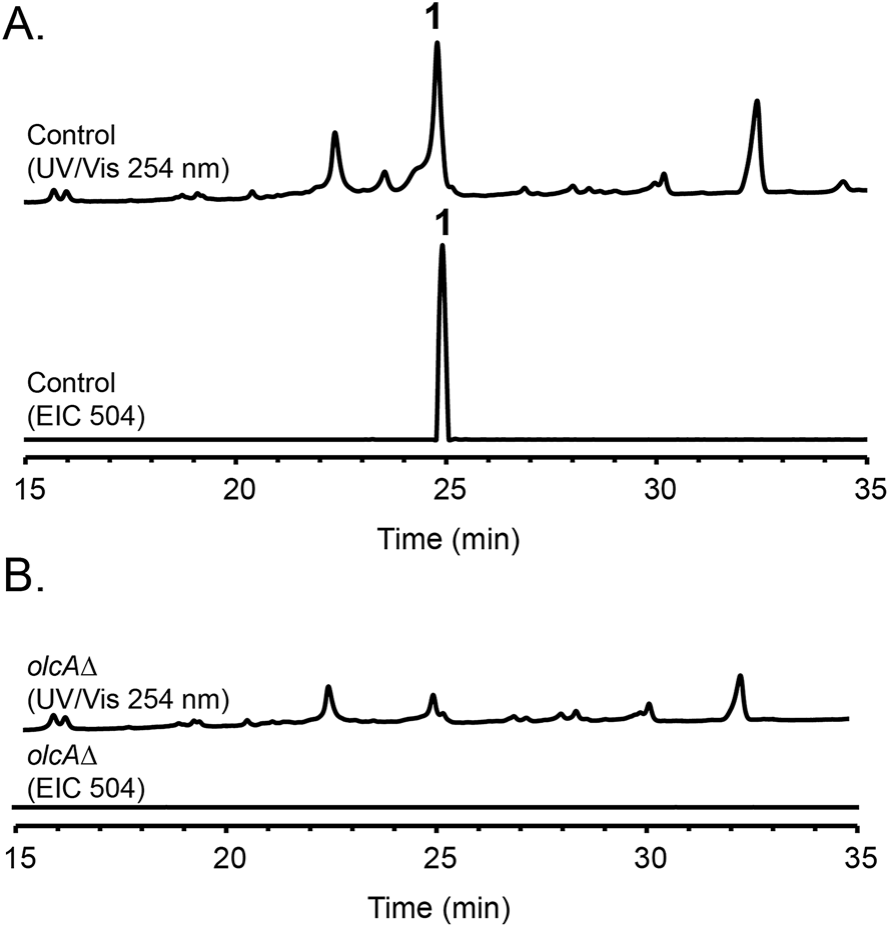
HPLC profiles of extracts from (A) parental strain and (B) *olcA*Δ strain as detected by UV-vis at 254 nm and mass spectrometry in positive mode of extracted ion chromatogram (EIC) at *m*/*z* = 504.

Fungal species are known to produce different secondary metabolites when cultivated on different culture media.^24^,^25^ We first grew *P. canescens* on 10 different solid culture media to see whether we were able to activate the pyripyropene A-like gene cluster and detect pyripyropene-like meroterpenoids. When grown on Czapek's medium, we found that compound **1** was produced in high titer (Fig. 2). LCMS analysis indicated that compound **1** has a molecular formula of C_30_H_32_NO_6_ and UV-vis maxima absorption at 238, 269 and 331 nm (Fig. S3†). The presence of a nitrogen atom as well as the similarities in UV-vis absorption patterns to pyripyropene A (232, 264 and 320 nm)^26^ indicated that compound **1** could be a pyripyropene-like meroterpenoid. For full characterization of compound **1**, the strain was subjected to large-scale cultivation, and the target peak was purified using flash chromatography and subsequently by preparative HPLC. Gratifyingly, the spectroscopic analysis revealed that compound **1** was 15-deoxyoxalicine B,^20^ a diterpenic meroterpenoid which belongs to a family of compounds that includes oxalicines and decaturins. To our knowledge, this is the first report of the production of this family of compounds in *P. canescens*. Furthermore, no molecular and genetic basis for the biosynthesis of oxalicines or decaturins has been reported previously.

### Development an efficient gene targeting system of *P*. *canescens* and identification of the gene cluster responsible for 15-deoxyoxalicine B biosynthesis

To confirm that protein ID 400488 (Pyr2 PKS homolog) in *P*. *canescens* ATCC 10419 is responsible for the biosynthesis of 15-deoxyoxalicine B, we next generated a protein ID 400488 deletant. For many fungal species, even if the complete genome sequence is available, linking SMs to specific genes is often challenging. This problem exists because for most of these organisms, gene targeting systems are not available or are inefficient, as was also the case for *P. canescens*. To develop an efficient gene targeting system for *P. canescens* that would facilitate the rapid generation of gene deletions, we created a *ku70Δ*, *pyrGΔ* double mutant strain of *P. canescens* ATCC 10419. The *ku70* gene was deleted to increase homologous recombination rates, thereby improving targeting efficiency.^27^,^28^ The gene *pyrG* was then deleted in the *ku70Δ* background to create an auxotrophic mutant that requires the supplementation of uracil and uridine.^29^ We next deleted the protein ID 400488 gene in a *P. canescens* ATCC 10419 strain with a *ku70Δ*, *pyrGΔ* background and cultivated the deletion strain under 15-deoxyoxalicine B-producing conditions. Analysis of the resultant SMs in the crude organic extract using LC-DAD-MS showed the complete elimination of compound **1** (Fig. 2). This result confirmed our hypothesis that protein ID 400488 is involved in the biosynthesis of **1**, and we designated this gene as *olcA*.

**Fig. 3:**
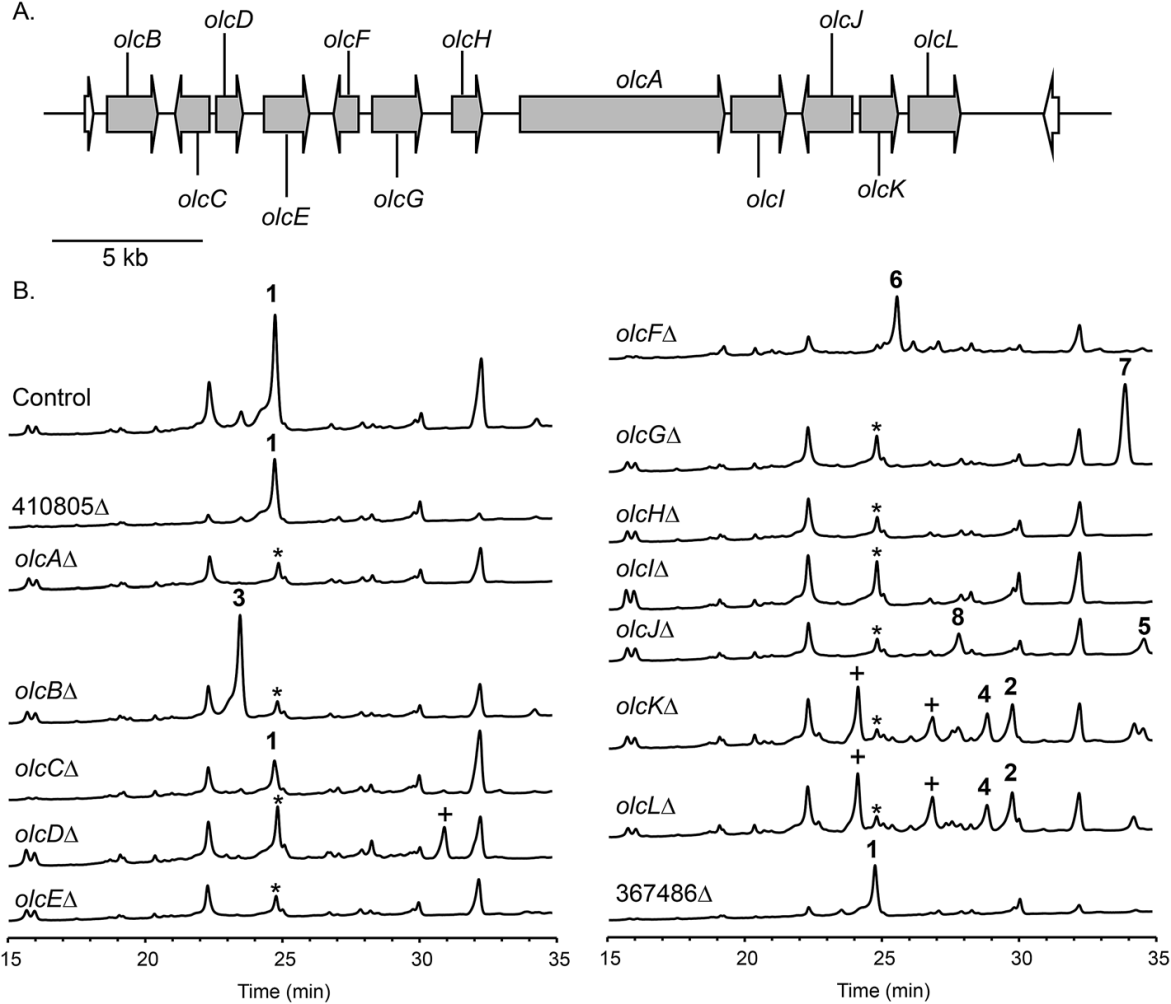
(A) Orientation of the genes surrounding the PKS *olcA* involved in 15-deoxyoxalicine B biosynthesis. Each arrow indicates gene size and direction of transcription. On the basis of a set of deletions we created and analyzed, genes shown in gray are responsible for 15-deoxyoxalicine B (**1**) biosynthesis while those in white are not. (B) HPLC extracts of strains in the cluster as detected by UV absorbance at 254 nm. A peak labeled by * appears at the same retention time as **1** in deletion strains. This compound was identified as griseofulvin. Peaks labeled with + denote compounds that could not be characterized because of poor yield or instability.

Next, we set out to identify additional genes involved in the biosynthesis of **1**. This process is facilitated by the fact that fungal secondary metabolite biosynthesis genes are usually clustered, and as mentioned previously, several *pyr* gene homologs were identified nearby (Fig. 3A and Table 1). In addition to these genes, we found that *olcA* is surrounded by genes that encode additional putative tailoring enzymes. We individually deleted 13 additional genes surrounding *olcA*. The 13 deletants were cultivated under 15-deoxyoxalicine B-producing conditions and their SM profiles were examined by LC-DAD-MS (Fig. 3B). Deletion of genes corresponding to protein IDs 333321, 437321, 351329, 367480, 393266, 410812, 437327, 333335, 367485, and 351342 resulted in complete elimination of **1**. Deletion of protein ID 351326 greatly diminished production of **1**. SM profiles remained unchanged after deletion of protein ID 410805 and 367486, indicating that these genes are not involved in the biosynthesis of **1** and that we have established the borders of the gene cluster. We now designate the genes surrounding *olcA* that are involved in the biosynthesis of **1** as *olcB*–*olcL* (Fig. 3A and Table 1).

### Identification, purification, and structural elucidation of the intermediates and shunt products from mutant strains

The deletant strains in which production of **1** was eliminated or greatly reduced were examined for the presence of new metabolites that may represent intermediates or shunt products of the biosynthetic pathway of **1**. Extracts from strains carrying deletions of *olcC*, *olcE*, *olcH*, and *olcI* showed no obvious intermediates that would be a part of the biosynthetic pathway of **1**. Six strains carrying gene deletions of six individual genes, *olcB*, *olcF*, *olcG*, *olcJ*, *olcK*, and *olcL* accumulated detectable intermediates or shunt products. These strains were subjected to large-scale cultivation and the target compounds were isolated by flash chromatography and semi-preparatory HPLC. Their structures were then elucidated by spectroscopic methods (NMR, MS, and UV-vis data for all the intermediates and shunt products identified are shown in ESI†). Among the isolated compounds were one oxalicine and four decaturin analogs: the *olcK* and *olcL* deletants yielded 15-deoxyoxalicine A (**2**), decaturin A (**3**)^20^ was isolated from *olcB* deletants, decaturin C (**4**)^21^ was isolated from both *olcK* and *olcL* deletants, decaturin D (**5**)^21^ was isolated from *olcJ* deletants, and decaturin F (**6**)^22^ was isolated from *olcF* deletants. Additionally, two new compounds, **7** and **8**, were isolated from *olcG* and *olcJ* deletants, respectively.

Compound **7** has a molecular formula of C_30_H_39_NO_4_ as determined by its ^13^C-NMR and HRESIMS data, representing 12 indices of hydrogen deficiency (IHD). The ^1^H, ^13^C, and gHSQC NMR data of **7** (Tables S5 and S6 and Fig. S6, S7 and S12†) exhibited signals for five methyl groups [*δ*_H_ 0.76, 0.87, 0.93, 1.03, and 1.66 (each 3H, s)], one carbinol methine group [*δ*_H_ 3.12 (1H, dd, *J* = 12.0 and 4.8 Hz, H-27); *δ*_C_ 79.9 (C-27)], one downfield methylene [*δ*_H_ 3.23 and 3.26 (each 1H, d, *J* = 16.2 Hz, H_2_-15); *δ*_C_ 24.0 (C-15)], and the pyridinyl-α-pyrone moiety [*δ*_H_ 6.65 (1H, s, H-12), 7.54 (1H, dd, *J* = 8.4 and 4.8 Hz, H-5), 8.23 (1H, dt, *J* = 8.4 and 1.8 Hz, H-4), 8.59 (1H, dd, *J* = 4.8 and 1.8 Hz, H-6), and 8.98 (1H, d, *J* = 1.8 Hz, H-2)]. Besides the pyridinyl-α-pyrone moiety, there are two additional quaternary sp^2^ carbons [*δ*_C_ 129.2 (C-16) and 138.3 (C-14)] (Table S6†). Considering that there are only 12 IHD in compound **7** with one pyridinyl-α-pyrone moiety and one olefin, compound **7** should contain a tricyclic diterpenoid moiety. Comparing the ^1^H and ^13^C NMR data of **7** with those of decaturin E (Tables S5 and S6†),^22^ the main differences are that there is no olefinic proton in the diterpenoid moiety at ∼5.7 ppm and no spiro carbon at ∼100.0 ppm in compound **7**, suggesting the existence of a tetrasubstituted olefin and absence of the spiro. In the gHMBC spectrum, the long-range ^1^H–^13^C correlations between H_2_-15 and three quaternary carbons (C-10, C-11, and C-14, Fig. S1a and S8–S11†) further connected the pyridinyl-α-pyrone and tricyclic diterpenoid moieties. Taken together, compound **7** was assigned to be a precursor before spiro formation (Scheme 1). Detailed analysis of gCOSY, gHSQC, and gHMBC NMR data (Fig. S1a and S8–S13†) allowed complete assignment of **7** and corroborated our proposed structure. We named compound **7** predecaturin E.

Compound **8**, which we named decaturin G, has a molecular formula of C_30_H_35_NO_5_. The ^1^H and ^13^C NMR spectra (Tables S4–S6 and Fig. S14 and S15†) indicated its structural resemblance to decaturin D (**5**), which was also isolated from the *olcJ* deletion strain. One main difference was the presence of an allylic carbinol methine proton [*δ*_H_ 4.46 (1H, br t, *J* = 4.8 Hz, H-18)] in **8**. The fact that H-19 is a doublet of doublets in **5**, and a doublet in **8** indicated that **8** is an 18-hydroxy derivative of **5**. A key NOE correlation between H-18 and H_eq_-25 indicated H-18 is an equatorial proton located at the α phase (Fig. S2†). gCOSY, gHSQC, and gHMBC NMR data (Fig. S1b and S16–S20†) allowed complete assignment of **8**.

## Discussion

We used a combination of genome mining, efficient gene targeting, and natural product chemistry to elucidate the biosynthetic pathway of the diterpenic meroterpenoid 15-deoxyoxalicine B (**1**). Several oxalicine analogues (oxalicines A and B) and decaturin analogues (decaturins A–F) have been isolated from *Penicillium* spp. such as *P. oxalicum*, *P. thiersii*, and *P. decateurense*,^18^–^23^,^30^ but this study is the first to our knowledge to give the molecular and genetic basis of the biosynthesis of this class of compounds.

On the basis of the structure similarity of the polyketide subunit with pyripyropenes, we performed bioinformatics analysis to find homologs of the responsible genes in *Penicillium* species. Using the genome sequence database provided by JGI, we found that *P. canescens* harbors homologs of all the genes in the pyripyropene A biosynthetic gene cluster. We then proceeded to perform a series of targeted gene deletions including genes encoding additional putative tailoring enzymes to identify the genes involved in the biosynthetic pathway of **1**. We showed that the pathway involves at least 12 genes in a single cluster. We have isolated and characterized 7 additional intermediates, 2 of which have not been reported previously. Further bioinformatics analysis together with the intermediates identified from the gene deletion strains has allowed us to propose a biosynthetic pathway for **1** (Scheme 1A).

Compound **1** has a polyketide subunit as a α-pyrone with an attached pyridine ring, very similar to pyripyropene A. This structural similarity has allowed us to propose the early steps of the biosynthetic pathway of **1** (Scheme 1A, blue). Although we did not detect intermediates from our LC-MS analysis of strains carrying deletions of *olcI* and *olcA*, these genes have high sequence similarity to genes involved in the first steps of the biosynthesis of pyripyropene A, *pyr1* and *pyr2*, respectively. This finding suggests that CoA ligase OlcI catalyzes the formation of nicotinyl-CoA. PKS OlcA then uses this nicotinyl-CoA as a starter unit to which it catalyzes the condensation of two malonyl-CoA molecules to form HPPO.

Unlike pyripyropene A in which a farnesyl pyrophosphate (FPP) is attached to HPPO in the subsequent step, **1** is linked with geranylgeranyl pyrophosphate (GGPP). The deletion of *olcC*, a gene for a GGPP synthase, significantly decreased the production of **1**, suggesting that a large portion of the GGPP used here seems to be generated by OlcC, however, the deletion did not completely eliminate the production of **1**. RT-PCR analysis confirmed the complete inactivation of *olcC* in the deletant strain (Fig. S5†). Bioinformatics analysis of the *P. canescens* genome showed 5 additional genes encoding GGPP synthases, which may provide the GGPP necessary to produce **1** in the *olcC* deletant strain. This *olcC* gene is interesting because, based on further bioinformatics analysis, this is the only GGPP synthase in *P. canescens* that is located in sufficient proximity to a PKS to be a part of a biosynthetic gene cluster. In contrast, the pyripyropene A gene cluster in *A. fumigatus* is located on a completely separate chromosome from the FPP synthase.

In pyripyropene A biosynthesis, the attachment of FPP to HPPO to form farnesyl-HPPO is catalyzed by the prenyltransferase Pyr6. The homolog of Pyr6 in *P. canescens* is OlcH (68% protein sequence similarity), and this enzyme is the most likely to catalyze the attachment of GGPP to HPPO to form geranylgeranyl–HPPO.

The next steps consist of the epoxidation and cyclization of the terpenoid subunit. It was demonstrated in the pyripyropene biosynthetic pathway that the FAD-dependent monooxygenase (FMO) Pyr5 catalyzes the epoxidation step, and the integral membrane protein Pyr4 functions as the terpene cyclase. The *olc* gene cluster also contains homologs of these two enzymes, OlcE (57% similarity) and OlcD (74% similarity). From this information, we propose that the FMO OlcE catalyzes the epoxidation of geranyl-geranyl–HPPO and OlcD catalyzes the cyclization of the terpenoid component, resulting in the formation of the tricyclic terpene moiety seen in predecaturin E (**7**).

Deletion of *olcG* resulted in the accumulation of **7**, suggesting that OlcG, a putative cytochrome P450, is the next enzyme in the biosynthetic pathway. We propose that OlcG catalyzes the allylic oxidation of compound **7**, which is followed by spirocylization with concomitant loss of one molecule of water to form decaturin E. Although our deletion strains did not produce decaturin E, we isolated and identified decaturin D (**5**) and decaturin G (**8**) from *olcJ* deletion strains. This result suggests that in the absence of OlcJ, decaturin E may be shunted to a pathway in which it is oxidized to a ketone, possibly by OlcF (see below), to form **5**, which undergoes further allylic oxidation to yield **8** (Scheme 1B).

The next steps involve the rearrangement of the diterpenic subunit leading to the formation of the hemiacetal seen in decaturin C (**4**) possibly *via* the 29-hydroxyl-27-one intermediate. Since compounds **5** and **8**, which accumulated in the *olcJ* deletion strain, both lack the 29-hydroxyl group, we propose that decaturin E is the substrate of the putative cytochrome P450 OlcJ which hydroxylates it at the C-29 position to form decaturin F (**6**). Conserved domain analysis showed OlcF is a putative short chain dehydrogenase, and deletion of *olcF* resulted in the accumulation of **6**. OlcF may catalyze the oxidation of **6** to generate the 29-hydroxyl-27-one intermediate, and subsequent hemiacetal formation will lead to the formation of **4**.

Deletion of *olcK* and *olcL* both resulted in similar SM profiles, showing the accumulation of **4**. This discovery suggests that OlcK and OlcL are downstream enzymes of **4**. *olcB* deletants accumulated decaturin A (**3**), which has an added hydroxyl group to **4**. These data suggest that both OlcK and OlcL are involved in the biotransformation of **4** to **3**. Conserved domain analysis of OlcK showed that this enzyme belongs to the 2-oxoglutarate-Fe(ii) oxygenase superfamily and has a 54% protein sequence similarity to Fum3, a fumonisin C-5 hydroxylase in *Fusarium verticillioides*.^31^ Interestingly, this enzyme superfamily includes peroxisomal enzymes. OlcL, on the other hand, is a putative MFS transporter. Computer analysis by TMHMM (; http://www.cbs.dtu.dk/services/TMHMM/) indicated that it is a highly hydrophobic protein with 14 transmembrane helices. Analysis of Pex19 (peroxisome biogenesis factor 19) binding sequences (; http://www.peroxisomedb.org/) in the OlcL protein revealed one putative Pex19 binding site between amino acids 132 and 143, within the 2^nd^ transmembrane helix. This finding suggests that OlcL may be inserted in the peroxisomal membrane *via* the import receptor Pex19. On the basis of these analyses, although speculative, we hypothesize that OlcK may be a peroxisomal enzyme that catalyzes the hydroxylation of **4** once it is shuttled into the peroxisome by the MFS transporter OlcL. However, localization studies will be necessary to test our hypothesis.

**Scheme 2:**
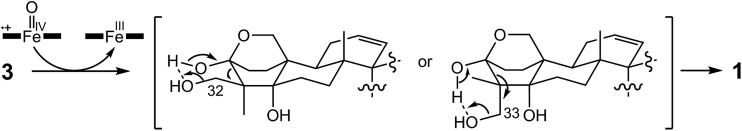
Proposed mechanism of oxidative rearrangement catalyzed by cytochrome P450 OlcB.

In the final step of 15-deoxyoxalicine B biosynthesis, the oxidative rearrangement^32^ of **3** could occur *via* either a 32- or 33-hydroxyl intermediate (Scheme 2). This reaction is catalyzed by a predicted cytochrome P450, OlcB, to yield **1**. In both *olcK* and *olcL* deletants, in addition to the production of **4**, we identified the production of 15-deoxyoxalicine A (**2**). On the basis of the function of OlcB, it is most likely that in the absence of OlcK and/or OlcL, **4** is accumulated and can be catalyzed by OlcB to yield **2** in a shunt pathway (Scheme 1B).

It is of note that the proposed 15-deoxyoxalicine B biosynthetic gene cluster does not contain a putative pathway-specific transcriptional activator gene. This is also the case for the previously reported pyripyropene biosynthetic gene cluster. This may indicate that the regulation of biosynthesis of these compounds are occurring at a more global level.

## Conclusion

Using a targeted gene-deletion approach, we have shown that a gene cluster consisting of 12 contiguous genes is involved in the biosynthesis of the diterpenic meroterpenoid 15-deoxyoxalicine B (**1**) in *P. canescens*. Combination of bioinformatics data and intermediates or shunt products isolated from the individual gene deletion mutants allowed us to propose a biosynthetic pathway for **1**.

## Materials and methods

### Strains and molecular genetic manipulations

The *P. canescens* wild-type and mutant strains used in this study are listed in Table S2.† All DNA insertions into the *P. canescens* genome were performed using protoplasts and standard PEG transformation. A strain of *P. canescens* ATCC 10419 was altered to improve gene targeting efficiency. First, the homolog of *ku70* was deleted by replacing it with the hygromycin resistance marker (*hph*). Hygromycin deletion cassettes were generated using the double joint PCR technique. Two ∼2000 base pair fragments upstream and downstream of the targeted gene were amplified from *P. canescens* genomic DNA by PCR. The two amplified flanking sequences and the hygromycin phosphor-transferase gene (*hph*) marker cassette amplified from pCB1003 (Fungal Genetics Stock Center) were fused together into one construct by fusion PCR using nested primers. Next, we created an auxotrophic mutant in the *ku70Δ* background by using a similar strategy, only this time we did not use a deletion cassette. We fused together the upstream and downstream fragments of *pyrG* from *P. canescens* to form the deletion construct. The mutation was selected by growth on media supplemented with uracil and fluoroorotic acid (FOA). FOA is toxic to cells that still have a functioning *pyrG* gene. Diagnostic PCR of the deletant strains was performed employing the external primers (P1 and P6) from the first round of PCR. The difference in size between the gene replaced by the selection marker and the native gene allowed us to determine whether the transformant carried the correct gene replacement. For transformants in which the size of the P1/P6 PCR products are similar to that of the control, additional diagnostic PCRs were carried out using external primers paired with primers located within the selection marker gene, in which case the deletants yielded PCR products of the expected size whereas no product would be seen in the non-deletants. All deletant strains were generated by replacing each targeted gene with the *P. canescens pyrG* gene (PcanpyrG) in the *ku70Δ*, *pyrG*Δ background strain of *P. canescens*.

### Fermentation and LC-MS analysis

Wild-type *P. canescens* ATCC 10419 and mutant strains were cultivated at 26 °C on Czapek's agar plates (complete medium; 3 g NaNO_3_ per L, 0.5 g KCl per L, 0.5 g MgSO_4_·7H_2_O per L, 0.01 g FeSO_4_·7H_2_O per L, 1 g K_2_HPO_4_ per L, 30 g sucrose per L, and agar 15 g L^–1^) starting with 1 × 10^7^ spores per Petri dish (*D* = 10 cm). After 5 days of cultivation, agar was chopped into small pieces and extracted by 80 ml MeOH followed by 80 ml 1 : 1 CH_2_Cl_2_/MeOH, each with 1 hour of sonication. The extract was evaporated *in vacuo* to yield a water residue, which was suspended in 50 ml H_2_O and partitioned with 50 ml EtOAc. The EtOAc layer was evaporated *in vacuo*, re-dissolved in 1 ml of 20% DMSO in MeOH, and a portion (10 μl) was examined by high performance liquid chromatography-photodiode array detection-mass spectroscopy (HPLC-DAD-MS) analysis.

HPLC-MS was carried out using a ThermoFinnigan LCQ Advantage ion trap mass spectrometer with a RP C18 column (Alltech Prevail C18 3 mm 2.1 × 100 mm) at a flow rate of 125 μl min^–1^. The solvent gradient for HPLC-DAD-MS was 95% MeCN/H_2_O (solvent B) in 5% MeCN/H_2_O (solvent A), both containing 0.05% formic acid, as follows: 0% solvent B from 0 to 5 min, 0–100% solvent B from 5 min to 35 min, 100–0% solvent B from 40 to 45 min, and re-equilibration with 0% solvent B from 45 to 50 min.

### Isolation and characterization of metabolites

For structure elucidation, the *P. canescens* wild-type and mutant strains were cultivated on ∼80 Czapek's agar plates (∼25 ml of medium per plate, *D* = 10 cm) at 1 × 10^7^ spores per plate at 26 °C for 6 days. Extraction was performed in the same manner as described above. The crude material was subjected to flash chromatography and further separated *via* semi-preparative reverse phase HPLC (Phenomenex Luna 5 μm C18 (2), 250 × 10 mm) with a flow rate of 5.0 ml min^–1^ and monitored by a UV detector at 235 nm. NMR spectra were collected on a Varian VNMRS-600 spectrometer. High-resolution electrospray ionization mass spectrum (HRESI-MS) was obtained with an Agilent Technologies 6210 time of flight mass spectrometer. Optical rotations were measured on a JASCO P-1010 digital polarimeter. The identity of previously reported compounds 15-deoxyoxalicine B, 15-deoxyoxalicine A, and decaturins A, C, D, and F (compounds **1–6**, respectively) were verified by HRESIMS, UV-vis, and ^1^H-NMR data (Tables S3 and S4†), which were in good agreement with previously published data.^20^–^22^ Details of large-scale purification of compounds from each strain and spectral data are provided in ESI.†

## Supplementary Material

Supplementary information
