# Correction: Genome mining and molecular characterization of the biosynthetic gene cluster of a diterpenic meroterpenoid, 15-deoxyoxalicine B, in *Penicillium canescens*

**DOI:** 10.1039/c6sc90012g

**Published:** 2016-02-03

**Authors:** Junko Yaegashi, Jillian Romsdahl, Yi-Ming Chiang, Clay C. C. Wang

**Affiliations:** a Department of Pharmacology and Pharmaceutical Sciences , School of Pharmacy , University of Southern California , Los Angeles , California 90089 , USA . Email: clayw@usc.edu; b Graduate Institute of Pharmaceutical Science , Chia Nan University of Pharmacy and Science , Tainan 71710 , Taiwan; c Department of Chemistry, College of Letters, Arts and Sciences , University of Southern California , Los Angeles , California 90089 , USA

## Abstract

Correction for ‘Genome mining and molecular characterization of the biosynthetic gene cluster of a diterpenic meroterpenoid, 15-deoxyoxalicine B, in *Penicillium canescens*’ by Junko Yaegashi *et al.*, *Chem. Sci.*, 2015, **6**, 6537–6544.



## 

The Joint Genome Institute and Dr. David Greenshields were inadvertently not acknowledged for allowing access to unpublished genome data. The following should be added to this paper as requested by the JGI:

We thank Dave Greenshields for providing access to *Penicillium canescens* unpublished genome data produced by the U.S. Department of Energy Joint Genome Institute, a DOE Office of Science User Facility, supported by the Office of Science of the U.S. Department of Energy under Contract No. DE-AC02-05CH11231.

The Royal Society of Chemistry apologises for these errors and any consequent inconvenience to authors and readers.

