# Supplementary material for: Genome mining and molecular characterization of the biosynthetic gene cluster of a diterpenic meroterpenoid, 15-deoxyoxalicine B, in Penicillium canescens
Source: Chem Sci. 2015 Aug 6;6(11):6537–44. doi: 10.1039/c5sc01965f (PMC6054112; doi:10.1039/c5sc01965f)
Supplement: Supplementary file 1 [file SC-006-C5SC01965F-s001.pdf]

Electronic Supplementary Material (ESI) for Chemical Science.
This journal is © The Royal Society of Chemistry 2015

Supporting Information

Genome Mining and Molecular Characterization of the Biosynthetic Gene Cluster of a Diterpenic Meroterpenoid, 15-Deoxyoxalicine B, in *Penicillium canescens*

Junko Yaegashi, Jillian Romsdahl, Yi-Ming Chiang, Clay C.C. Wang

Correspondence should be addressed to C. C. C. W (clayw@usc.edu)

Table of Contents

Supplemental Methods **S2**

Spectral Data of compounds **S2**

Table S1. Primers used in this study **S5**

Table S2. *P. canescens* strains used in this study **S8**

Table S3. ^{1}H-NMR data for compounds **1**, **2** and **3** **S9**

Table S4. ^{1}H-NMR data for compounds **4, 5** and **6** **S10**

Table S5. ^{1}H-NMR data for compounds **7** and **8** **S11**

Table S6. ^{13}C-NMR data for compounds **7** and **8** **S12**

Figure S1. Key gHMBC correlations of compounds **7** and **8** **S13**

Figure S2. Selective 1D NOESY correlations of compound **8** **S13**

Figure S3. UV-Vis and ESIMS spectra of compounds **1** to **8** **S14**

Figure S4. Results of diagnostic PCR for all the gene deletion strains **S16**

Figure S5. Result of RT-PCR analysis for *olcC* **S17**

Figure S6-S20. NMR spectra of compounds **7** and **8** **S18-S32**

Supplemental Methods

Isolation and identification of secondary metabolites

All strains were cultivated at 26°C for 6 days on ~200 CZA plates at 10 x 10^6 spores per 10 cm plate. Similar to the method described above, the agar was chopped and sonicated in MeOH, followed by 1:1 CH_2Cl_2/MeOH. The organic material was evaporated and extracted twice with an equal volume of EtOAc. All EtOAc layers were combined and evaporated *in vacuo*.

For isolation of 15-deoxyoxalicine B and its biosynthetic intermediates, the crude extract in the EtOAc layer (~150 mg) was coated on 2.3 g C_{18} silica gel (Cosomil 75C_{18}-OPN, Nacalai Tesque), which was then suspended in MeOH and applied to a silica gel column (32 x 50 mm). After equilibrating the column to the starting solvent system of 1:9 MeOH-H_2O, the extract was eluted with MeOH-H_2O mixtures of decreasing polarity (fraction A, 1:9, 150 mL; fraction B, 1:1, 150 mL; fraction C, 3:1, 150 mL; fraction D, 1:0, 150 mL). All fractions were analyzed by HPLC-DAD-MS. Fraction C was subjected to semi-preparative reverse phase HPLC (Phenomenex Luna 5 μm C18 (2), 250 x 10 mm) with a flow rate of 5.0 ml/min and monitored by a UV detector at 235 nm. The gradient system was MeCN (solvent B) in 5% MeCN/H_2O (solvent A): 30 to 100% solvent B from 0 to 35 min, maintained at 100% from 35 to 38 min, 100 to 30% solvent B from 38 to 39 min, and re-equilibration with 30% solvent B from 39 to 43 min. Compounds **1** (1.8 mg), **2** (0.9 mg), **3** (1.2 mg), **4** (0.9 mg), **5** (0.8 mg), **6** (1.0 mg), **7** (1.8 mg), and **8** (0.8 mg) eluted at 9 min, 19 min, 8 min, 15 min, 22 min, 22 min, 20 min, and 14 min, respectively.

RT-PCR analysis of the expression of *olcC*

The *P. canescens* wild type and the *olcC* deletant strain were cultivated on LCMM agar at 26 °C for 6 days for mRNA extraction. The β-tubulin gene (protein ID 352267) was used as a control and quantification standard. Total mRNA was extracted by using the Qiagen RNeasy Plant Mini Kit. The total mRNA was digested by Recombinant DNase I (Ambion) to remove DNA contamination. The cDNA library was made from the same amount of total mRNA by using TaqMan reverse transcription reagents (Applied Biosystems) and the oligo dT primer. The cDNA was then used as the template for PCR amplification with specific primer sets listed in Table S1. Amplification products were analyzed by electrophoresis in 1% agarose gels stained with ethidium bromide.

Spectral data of Compounds

For compound structure elucidation, ^{1}H and ^{13}C spectra were collected on nuclear magnetic resonance (NMR) Varian VNMRS-600 and Varian Mercury Plus 400 spectrometers. High-resolution electrospray ionization mass spectrum (HRESI-MS) was obtained with an Agilent Technologies 6210 time of flight mass spectrometer. Optical rotations were measured with a JascoP-2000 polarimeter operating on the sodium D-line (589 nm), using a 100 mm path-length and are reported as $[\alpha]_D^T$ (concentration in g/100 mL, solvent).

15-deoxyoxalicine B (1)
White amorphous powder; $[\alpha]_D^{25}$: + 47.3 (*c* 0.15, CH_2Cl_2).
UV/Vis λ_{max} $(MeOH)_{max}$: 238, 269, 331 nm.
^1H NMR: Table S3.
HRESI-MS, $[M + H]^+$ *m/z* found 504.2374 calc. for $C_{30}H_{33}NO_6$: 504.2381.

15-deoxyoxalicine A (2)
White amorphous powder.
UV/Vis λ_{max} $(MeOH)_{max}$: 238, 269, 331 nm.
^1H NMR: Table S3.
HRESI-MS, $[M + H]^+$ *m/z* found 488.2434 calc. for $C_{30}H_{33}NO_5$: 488.2431.

Decaturin A (3)
White amorphous powder; $[\alpha]_D^{25}$: + 73.0 (*c* 0.10, CH_2Cl_2).
UV/Vis λ_{max} $(MeOH)_{max}$: 238, 269, 333 nm.
^1H NMR: Table S3.
HRESI-MS, $[M + H]^+$ *m/z* found 506.2546 calc. for $C_{30}H_{35}NO_6$: 506.2537.

Decaturin C (4)
White amorphous powder; $[\alpha]_D^{25}$: + 86.7 (*c* 0.08, CH_2Cl_2).
UV/Vis λ_{max} $(MeOH)_{max}$: 238, 264, 331 nm.
^1H NMR: Table S4.
HRESI-MS, $[M + H]^+$ *m/z* found 490.2591 calc. for $C_{30}H_{35}NO_5$: 490.2588.

Decaturin D (5)
White amorphous powder; $[\alpha]_D^{25}$: + 85.1 (*c* 0.07, CH_2Cl_2).
UV/Vis λ_{max} $(MeOH)_{max}$: 236, 264, 331 nm.
^1H NMR: Table S4.
HRESI-MS, $[M + H]^+$ *m/z* found 474.2617 calc. for $C_{30}H_{35}NO_4$: 474.2639.

Decaturin F (6)
White amorphous powder; $[\alpha]_D^{25}$: + 92.8 (*c* 0.08, MeOH).
UV/Vis λ_{max} $(MeOH)_{max}$: 238, 269, 331 nm.
^1H NMR: Table S4.
HRESI-MS, $[M + H]^+$ *m/z* found 492.2752 calc. for $C_{30}H_{37}NO_5$: 492.2751.

Predecaturin E (7)
White amorphous powder; $[\alpha]_D^{25}$: - 48.7 (*c* 0.15, MeOH).
UV/Vis λ_{max} $(MeOH)_{max}$: 235, 331 nm.
^1H NMR: Table S5 and Figure S6; ^{13}C NMR: Table S6 and Figure S7.
HRESI-MS, $[M + H]^+$ *m/z* found 478.2962 calc. for $C_{30}H_{39}NO_4$: 478.2952.

Decaturin G (8)
White amorphous powder.
UV/Vis λ_{max} $(MeOH)_{max}$: 238, 269, 333 nm.

^{1}H NMR: Table S5 and Figure S14; ^{13}C NMR: Table S6 and Figure S15.
HRESI-MS, $[M + H]^+$ *m/z* found 490.2568 calc. for $C_{30}H_{35}NO_5$: 490.2588.

Table S1. Primers used in this study (5’ → 3’)

410805-P1	TTGGCTGGATCGGTGATT
410805-P2	AGCGGACGATTTTTGCTG
410805-P3	CGAAGAGGGTGAAGAGCATTGCAGCGCATAAACGCATTG
410805-P4	CAGTGCCTCCTCTCAGACAGCCTAAACACCACGCAAAGG
410805-P5	TGTGGCATCACAGCAAGG
410805-P6	ATCCTGGGCGATTGAGG
olcA deletion	
400488-P1	AACGACCCGCATACTGGA
400488-P2	CTCAGGCCACGAATACGC
400488-P3	CGAAGAGGGTGAAGAGCATTGACGGAACTGGTGGGGAAC
400488-P4	CAGTGCCTCCTCTCAGACAGTCAAGCCCACTTCCAAGG
400488-P5	CCCAGAGTTGTCCGATGC
400488-P6	GGTTGTCCCATCGTCCAG
olcB deletion	
333321-P1	CGCATGTGGCTGTACTCG
333321-P2	CGTTTATGCGCTGGCTTT
333321-P3	CGAAGAGGGTGAAGAGCATTGTCGGAGCCTGAAGTCGTC
333321-P4	CAGTGCCTCCTCTCAGACAGTATGGAACACCCCGCAGT
333321-P5	GTCCGACCGAGGAGGAAT
333321-P6	AAGGGTCAGGGCATGGAT
olcC deletion	
351326-P1	GGATGGTTGGGTAGCTCGT
351326-P2	ACATTGTGGGCAAACATGG
351326-P3	CGAAGAGGGTGAAGAGCATTGAACTGCGATCCGCATCAT
351326-P4	CAGTGCCTCCTCTCAGACAGCCCATCCTTTGCATGGTC
351326-P5	CATTCCGCCCAGAGTCAG
351326-P6	GCATGAGTCCCGATACGC
olcD deletion	
437321-P1	TTGGATTCCCGCTGTTTG
437321-P2	TCAGAATTGCTGCGGATG
437321-P3	CGAAGAGGGTGAAGAGCATTGAAGGTGAAGGGCCGACTC
437321-P4	CAGTGCCTCCTCTCAGACAGCGAGCTACCCAACCATCC
437321-P5	TCGCCACATTTCTGTTCG
437321-P6	GCACAGCAGCAGAAATGC
olcE deletion	
351329-P1	TTCTGCACTGCGATTTGC
351329-P2	CTTGCTGTCGGGTTCTGG
351329-P3	CGAAGAGGGTGAAGAGCATTGGTAAAAGCCGGGTTGTGG
351329-P4	CAGTGCCTCCTCTCAGACAGACAGTTCGACGTAGCCTTGG

351329-P5	GGCAAGCATGGTTTGATAGG
351329-P6	CCGCAAGTAAGTCCATACCC

olcF deletion

367480-P1	CGATCTTGCGAGCTTTCC
367480-P2	CTCGGAAGGCAAGGACTG
367480-P3	CGAAGAGGGTGAAGAGCATTGCGGCTCGGATCGAAGTAG
367480-P4	CAGTGCCTCCTCTCAGACAGATCACCGCCCTGTTTGAC
367480-P5	AGGGCCAGGTTGGATCTC
367480-P6	ACCAAGCCTCACGTCTCG

olcG deletion

393266-P1	CTTTGATCGGAGGCCAAG
393266-P2	TGCCTCGTGATCGAATTG
393266-P3	CGAAGAGGGTGAAGAGCATTGGCCCGCAGGTGAAGTATG
393266-P4	CAGTGCCTCCTCTCAGACAGCCAGCACAGGGAAGAACC
393266-P5	ACCCAGTCGTTCCACACC
393266-P6	TGTACGCGCCACTTTGG

olcH deletion

410812-P1	CCTCGGCTAACCAGTGGA
410812-P2	GCCATTTACCCCGATCCT
410812-P3	CGAAGAGGGTGAAGAGCATTGTTGGCGGAGAATTGGAAA
410812-P4	CAGTGCCTCCTCTCAGACAGAAATGGGGATGGCTCGAT
410812-P5	CCAAGGCACCACATCCTT
410812-P6	CGCGTACTGGGGAGTGAA

olcI deletion

437327-P1	ACGCCATTTCTGGACACC
437327-P2	GGACTGGACCGCATCAAA
437327-P3	CGAAGAGGGTGAAGAGCATTGTGATCCGCTCAGCATGAA
437327-P4	CAGTGCCTCCTCTCAGACAGTCCGATTTTGGGGGAAAC
437327-P5	CGCCATTCACACAACGAC
437327-P6	CTACTGGGCGGTTCATCG

olcJ deletion

333335-P1	TCCATCCTCCCGTCTCTG
333335-P2	GCCGGGTGCGTAGTTATG
333335-P3	CGAAGAGGGTGAAGAGCATTGGGCGCATCAATCATTTC
333335-P4	CAGTGCCTCCTCTCAGACAGTTGTTTCCCCCAAAATCG
333335-P5	AGCGATGCCAGAAGTTGC
333335-P6	AGTCCACCCTCCCTGTCC

olcK deletion

367485-P1	GTACGCCACAGCCATCG
367485-P2	GGCCACTATGGGGTGTAGG

367485-P3	CGAAGAGGGTGAAGAGCATTGTGGAGAAGCACGGAAAGG
367485-P4	CAGTGCCTCCTCTCAGACAGAACCTTGGTCACCCATCG
367485-P5	CCAACGTCTCCCAACGAG
367485-P6	TAGGGAGCTGGGTGTTGC

olcL deletion

351342-P1	AAGCTGTTCCGTGCCAAC
351342-P2	TTGATTCCGGCGAAAAAG
351342-P3	CGAAGAGGGTGAAGAGCATTGGGACGGATTGTCCTGTCG
351342-P4	CAGTGCCTCCTCTCAGACAGCAATTGGCCGGATACAGG
351342-P5	TGGCCCGAGTTATCTAGTCG
351342-P6	GCCCACATCCCCTTACC

367486-P1	AAGTGCCTCGCATCCTGC
367486-P2	TAAGGGGATGTGGGCGGA
367486-P3	CGAAGAGGGTGAAGAGCATTGGGGTCCGCTCAGGGTAGA
367486-P4	CAGTGCCTCCTCTCAGACAGCAGATGTTCGCCCGGGTT
367486-P5	CGAACCGTGCAGGTGGAA
367486-P6	GCCTCACACACGTGCTCA

olcC RT-PCR

351326-Fw	GGTCAGGGCATGGATCTCTA
351326-Rev	CCTCGGACTTCTGTTTCAGG

β-tubulin RT-PCR

352267-Fw	ATGGGCACACTCCTGATCTC
352267-Rev	CGACCATGAAGAAGTGCAGA

Blue and red sequences are tails that anneal to the *P. canescens* pyrG fragment (PcanpyrG) during fusion PCR.

Table S2. *Penicillium canescens* strains used in this study.

Label	Genotype
Control	*ku70::hph*
*ku70*Δ, *pyrG*Δ	*ku70::hph; pyrG-*
410805Δ	*ku70::hph; pyrG-*, ProteinID410805*::PcanpyrG*
*olcA*Δ	*ku70::hph; pyrG-*, ProteinID400488*::PcanpyrG*
*olcB*Δ	*ku70::hph; pyrG-*, ProteinID333321*::PcanpyrG*
*olcC*Δ	*ku70::hph; pyrG-*, ProteinID351326*::PcanpyrG*
*olcD*Δ	*ku70::hph; pyrG-*, ProteinID437321*::PcanpyrG*
*olcE*Δ	*ku70::hph; pyrG-*, ProteinID351329*::PcanpyrG*
*olcF*Δ	*ku70::hph; pyrG-*, ProteinID367480*::PcanpyrG*
*olcG*Δ	*ku70::hph; pyrG-*, ProteinID393266*::PcanpyrG*
*olcH*Δ	*ku70::hph; pyrG-*, ProteinID410812*::PcanpyrG*
*olcI*Δ	*ku70::hph; pyrG-*, ProteinID437327*::PcanpyrG*
*olcJ*Δ	*ku70::hph; pyrG-*, ProteinID333335*::PcanpyrG*
*olcK*Δ	*ku70::hph; pyrG-*, ProteinID367485*::PcanpyrG*
*olcL*Δ	*ku70::hph; pyrG-*, ProteinID351342*::PcanpyrG*
367486Δ	*ku70::hph; pyrG-*, ProteinID367486*::PcanpyrG*

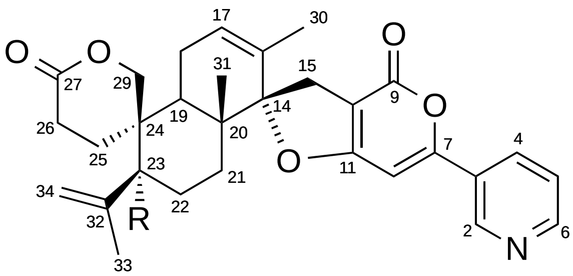

1: R = OH
2: R = H

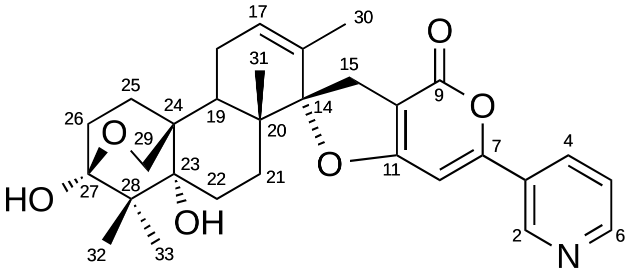

3

Table S3. ^{1}H-NMR data for compounds **1**, **2**, and **3** (600 MHz in $CDCl_3$)[a]

position	1	2	3
**2**	8.99 (1H, d, 1.8)	9.00 (1H, d, 1.8)	9.00 (1H, d, 1.8)
**4**	8.11 (1H, dt, 8.4, 1.8)	8.12 (1H, dt, 8.4, 1.8)	8.10 (1H, dt, 8.4, 1.8)
**5**	7.39 (1H, dd, 8.4, 4.8)	7.40 (1H, dd, 8.4, 4.8)	7.39 (1H, dd, 8.4, 4.8)
**6**	8.66 (1H, dd, 4.8, 1.8)	8.67 (1H, dd, 4.8, 1.8)	8.67 (1H, dd, 4.8, 1.8)
**12**	6.64 (1H, s)	6.62 (1H, s)	6.61 (1H, s)
**15**	2.97 (1H, d, 16.8)	2.96 (1H, d, 16.2)	2.94 (1H, d, 16.2)
	3.10 (1H, d, 16.8)	3.09 (1H, d, 16.2)	3.08 (1H, d, 16.2)
**17**	5.72 (1H, br s)	5.72 (1H, br s)	5.69 (1H, br d, 5.4)
**18**	2.21 (2H, m)	2.25 (2H, m)	1.87 (1H, m)
			2.06 (1H, m)
**19**	2.67 (1H, dd, 11.4, 6)	1.99 (1H, m)	2.24 (1H, dd, 12.6, 4.8)
**21**	1.49 (1H, dt, 14.4, 3.6, H_{eq})	1.56 (2H, m)	1.29 (1H, dt, 13.8, 3.6, H_{eq})
	2.30 (1H, m, H_{ax})		1.84 (1H, td; 13.8, 3.6, H_{ax})
**22**	1.30 (1H, dt, 13, 3.6, H_{eq})	1.56 (1H, m)	1.48 (1H, dt, 14.4, 3.6, H_{eq})
	2.05 (1H, td, 13, 4.2, H_{ax})	1.76 (1H, m)	2.10 (1H, td, 14.4, 3.6, H_{ax})
**23**		2.01 (1H, br s)	
**25**	1.62 (1H, m)	1.76 (1H, m)	1.72 (1H, m, H_{β})
	2.49 (1H, m)	2.25 (1H, m)	1.93 (1H, td, 12, 7.2, H_{α})
**26**	2.33 (1H, m)	2.40 (1H; m)	1.65 (1H, m)
	2.43 (1H, m)	2.44 (1H, m)	2.22 (1H, m)
**29**	4.37 (1H, d, 12.9)	4.37 (1H, d, 12)	3.93 (1H, d, 9.6)
	4.45 (1H, d, 12.9)	4.53 (1H, d, 12)	4.12 (1H, dd, 9.6, 2.4)
**30**	1.70 (3H, s)	1.70 (3H, s)	1.67 (3H, s)
**31**	0.92 (3H, s)	0.95 (3H, s)	0.87 (3H, s)
**32**			1.00 (3H, s)
**33**	5.07 (1H, s)	4.76 (1H, s)	1.07 (3H, s)
	5.17 (1H, s)	4.96 (1H, s)	
**34**	1.89 (3H, s)	1.80 (3H, s)	

[a] Figures in parentheses are multiplicities, and coupling constants (*J*) in Hz

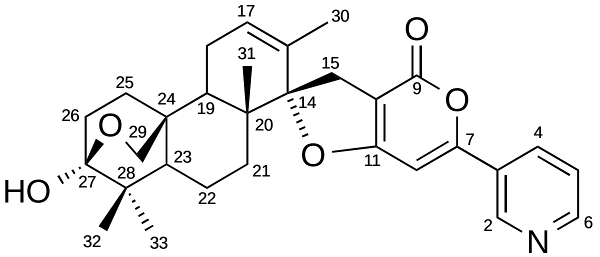

4

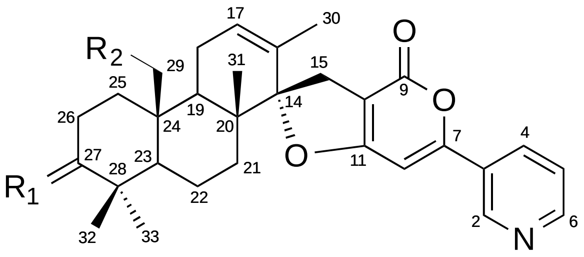

5: R_1= O; R_2 = H
6: R_1 = β-OH, α-H; R_2 = OH

Table S4. ^{1}H-NMR data for compounds **4**, **5** (600 MHz in $CDCl_3$), and **6** (600 MHz in DMSO-*d_6*)[a]

position	4	5	6
**2**	9.00 (1H, d, 1.8)	9.01 (1H, d, 1.8)	9.07 (1H, d, 1.8)
**4**	8.12 (1H, dt, 8.4, 1.8)	8.12 (1H, dt, 8.4, 1.8)	8.24 (1H, dt, 8.4, 1.8)
**5**	7.39 (1H, dd, 8.4, 4.8)	7.39 (1H, dd, 8.4, 4.8)	7.54 (1H, dd, 8.4, 4.8)
**6**	8.67 (1H, dd, 4.8, 1.8)	8.66 (1H, dd, 4.8, 1.8)	8.67 (1H, 4.8, 1.8)
**12**	6.60 (1H, s)	6.63 (1H, s)	7.36 (1H, s)
**15**	2.92 (1H, d, 16.2)	2.95 (1H, d, 16.2)	2.88 (1H, d, 16.2)
	3.08 (1H, d, 16.2)	3.10 (1H, d, 16.2)	3.04 (1H, d, 16.2)
**17**	5.70 (1H, br d, 5.4)	5.71 (1H, br s)	5.65 (1H, br d, 4.8)
**18**	1.80 (1H, m)	2.07 (2H, m)	2.06 (1H, m)
	2.14 (1H, m)		2.63 (1H, m)
**19**	1.80 (1H, m)	1.77 (1H, dd, 10.2, 6.6)	1.58 (1H, m)
**21**	1.35 (1H, m)	1.41-1.46 (1H, m)	1.35 (1H, dd, 12.6, 4.2)
	1.53 (1H, m)	1.50-1.60 (1H, m)	1.59 (1H, m)
**22**	1.56 (2H, m)	1.50-1.60 (1H, m)	1.42-1.46 (2H, m)
**23**	1.29 (1H, m)	1.41-1.46 (1H, m)	0.78 (1H, dd, 13.8, 2.4)
**25**	1.27 (1H, m)	1.50-1.60 (1H, m)	0.68 (1H, 13.2, 3.0)
	2.16 (1H, m)	1.93 (1H, m)	2.24 (1H, 13.2, 3.0)
**26**	1.73 (1H, m)	2.42 (1H, m)	1.42-1.46 (1H, m)
	2.20 (1H, m)	2.55 (1H, m)	1.56 (1H, m)
**27**			3.02 (1H, dd, 12, 4.8)
**29**	3.89 (1H, dd, 9, 1.8)	1.071 (3H, s)	3.79 (1H, d, 12)
	4.20 (1H, dd, 9, 3)		3.76 (1H, d, 12)
**30**	1.66 (3H, br s)	1.69 (3H, br d, 1.8)	1.61 (3H, s)
**31**	0.87 (3H, s)	0.97 (3H, s)	1.02 (3H, s)
**32**	0.97 (3H, s)	1.05 (3H, s)	0.71 (3H, s)
**33**	1.03 (3H, s)	1.074 (3H, s)	0.89 (3H, s)

[a] Figures in parentheses are multiplicities and coupling constants (*J*) in Hz

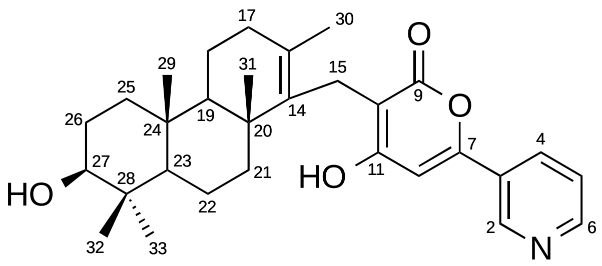

7

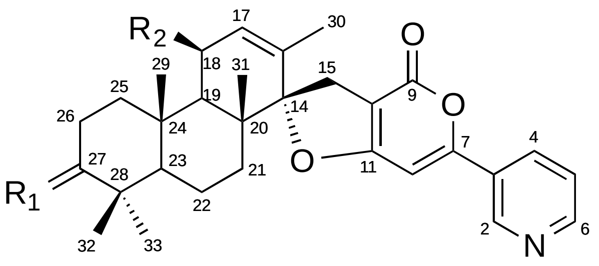

8: R_1 = O; R_2 = OH
Decaturin E: R_1 = β-OH, α-H; R_2 = H

Table S5. ^{1}H-NMR Data for Compounds **7** and **8** (600 MHz in CD_3OD) compared to previously published data of structurally related Decaturin E (600 MHz in DMSO-d_6)[a, b]

position	Decaturin E	7	8
**2**	9.07 (1H, d, 1.9)	8.98 (1H, d, 1.8)	9.06 (1H, dd, 1.8, 0.9)
**4**	8.23 (1H, ddd, 8.2, 1.9, 1.4)	8.23 (1H, dt, 8.4, 1,8)	8.30 (1H, dt, 8.4, 1.8)
**5**	7.54 (1H, dd, 8.2, 4.7)	7.54 (1H, dd, 8.4, 4.8)	7.57 (1H, dd, 8.4, 4.8)
**6**	8.67 (1H, dd, 4.7, 1.4)	8.59 (1H, dd, 4.8, 1.8)	8.64 (1H, dd, 4.8, 1.8)
**12**	7.36 (1H, s)	6.65 (1H, s)	7.05 (1H, s)
**15**	2.85 (1H, d, 16.1)	3.25 (1H, d, 16.2)	3.07 (1H, d, 16.2)
	3.01 (1H, d, 16.1)	3.24 (1H, d, 16.2)	3.13 (1H, d, 16.2)
**17**	5.66 (1H, br s)	2.01-2.12 (2H, m)	5.75 (1H, br d, 4.8)
**18**	1.98 (2H, m)	1.46-1.51 (1H, m)	4.46 (1H, br t, 4.8)
		1.50-1.60 (1H, m)	
**19**	1.58 (1H, m)	1.11 (1H, dd, 12.6, 1.8)	1.70 (1H, d, 4.8)
**21**	1.28 (1H, m)	1.34 (1H, td, 13.2, 3.6), H_{ax}	1.51-1.67 (2H, m)
	1.56 (1H, m)	2.01-2.12 (1H, m), H_{eq}	
**22**	1.41 (1H, m)	1.43 (1H, td, 13.2, 1.8), H_{ax}	1.51-1.67 (2H, m)
	1.52 (1H, m)	1.50 -1.60 (1H, m), H_{eq}	
**23**	0.70 (1H, m)	0.78 (1H, dd, 12.0, 1.8)	1.36 (1H, br d, 12)
**25**	0.91 (1H, m)	0.96 (1H, td, 13.2, 3.6), H_{ax}	1.51-1.67 (1H, m)
	1.60 (1H, m)	1.78 (1H, dt, 13.2, 3.6), H_{eq}	2.37 (1H, m)
**26**	1.48 (2H, m)	1.59-1.63 (1H, m)	2.37 (1H, m)
		1.63-1.69 (1H, m)	2.75 (1H, m)
**27**	2.98 (1H, m)	3.13 (1H, dd, 12, 4.8)	
**29**	0.89 (3H, s)	0.87 (3H, s)	1.56 (3H, s)
**30**	1.62 (3H, s)	1.66 (3H, s)	1.78 (3H, s)
**31**	0.87 (3H, s)	1.03 (3H, s)	1.30 (3H, s)
**32**	0.68 (3H, s)	0.76 (3H, s)	1.10 (3H, s)
**33**	0.86 (3H, s)	0.93 (3H, s)	1.05 (3H, s)

[a] Figures in parentheses are multiplicities and coupling constants (*J*) in Hz
[b] Ref: Wang, P.-l.; Li, D.-y.; Xie, L.-r.; Wu, X.; Hua, H.-m.; Li, Z.-l. *Nat. Prod. Commun.* **2013**, *8*, 1397-1398.

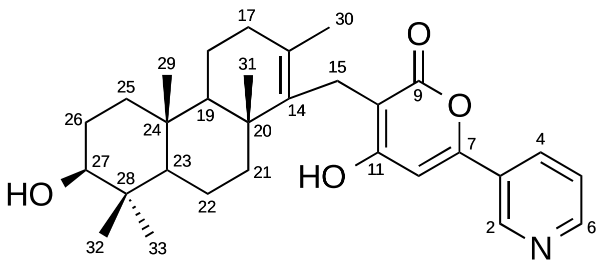

7

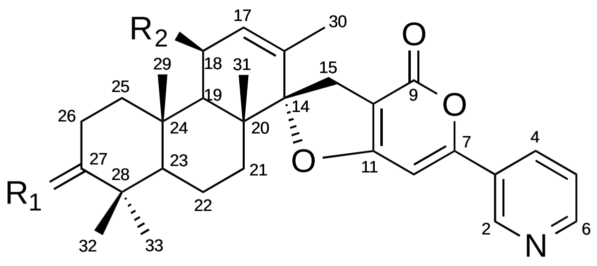

8: R_1 = O; R_2 = OH
Decaturin E: R_1 = β-OH, α-H; R_2 = H

Table S6. ^{13}C-NMR data for compounds **7** and **8** (150 MHz in CD_3OD) compared to previously published data of structurally related Decaturin E (150 MHz in DMSO-*d*$_6$)[a]

position	Decaturin E	7	8
**2**	146.7	147.1	147.8
**3**	127.2	129.8	129.2
**4**	133.1	134.8	135.3
**5**	124.0	125.6	125.6
**6**	151.4	151.5	152.2
**7**	159.5	155.9	161.6
**9**	170.0	167.2	172.8
**10**	101.2	102.7	103.4
**11**	159.6	179.7	163.0
**12**	94.2	106.0	95.8
**14**	100.1	138.3	102.3
**15**	27.8	24.0	29.8
**16**	130.9	129.2	133.5
**17**	128.1	35.7	132.1
**18**	22.5	19.4	65.3
**19**	47.4	57.9	52.0
**20**	40.3	40.7	41.9
**21**	31.7	39.7[b]	33.5
**22**	17.5	19.8	20.6
**23**	54.5	56.9	57.5
**24**	36.4	38.5	39.0
**25**	37.9	39.9[b]	39.6[c]
**26**	26.9	28.2	35.1
**27**	76.8	79.9	218.5
**28**	38.4	40.1	39.5[c]
**29**	15.4	17.2	17.6
**30**	18.1	21.0	19.0
**31**	15.9	22.0	19.7
**32**	16.0	16.2	22.4
**33**	28.2	28.7	26.9

[a] Ref: Wang, P.-l.; Li, D.-y.; Xie, L.-r.; Wu, X.; Hua, H.-m.; Li, Z.-l. *Nat. Prod. Commun.* **2013**, *8*, 1397-1398.

[b, c] These assignments may be interchanged.

(a)

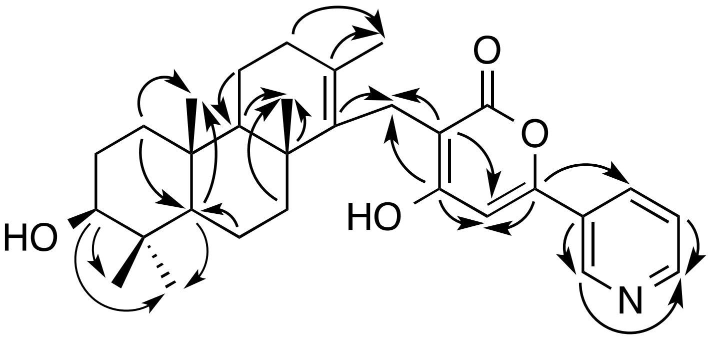

(b)

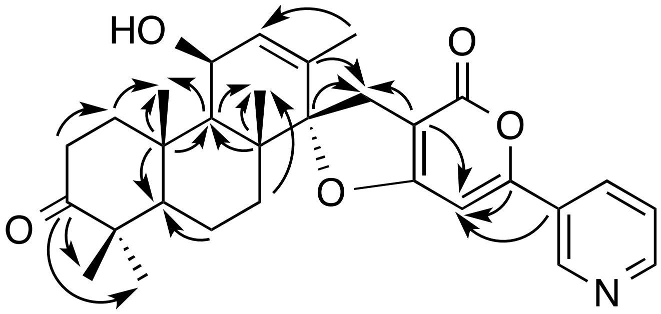

Figure S1. Key gHMBC correlations (C→H) of (a) predecaturin E (**7**) and (b) decaturin G (**8**).

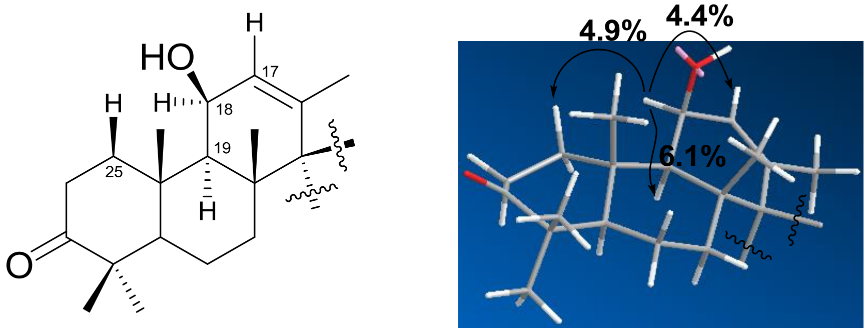

Figure S2. Selective 1D NOESY correlations of decaturin G (**8**).

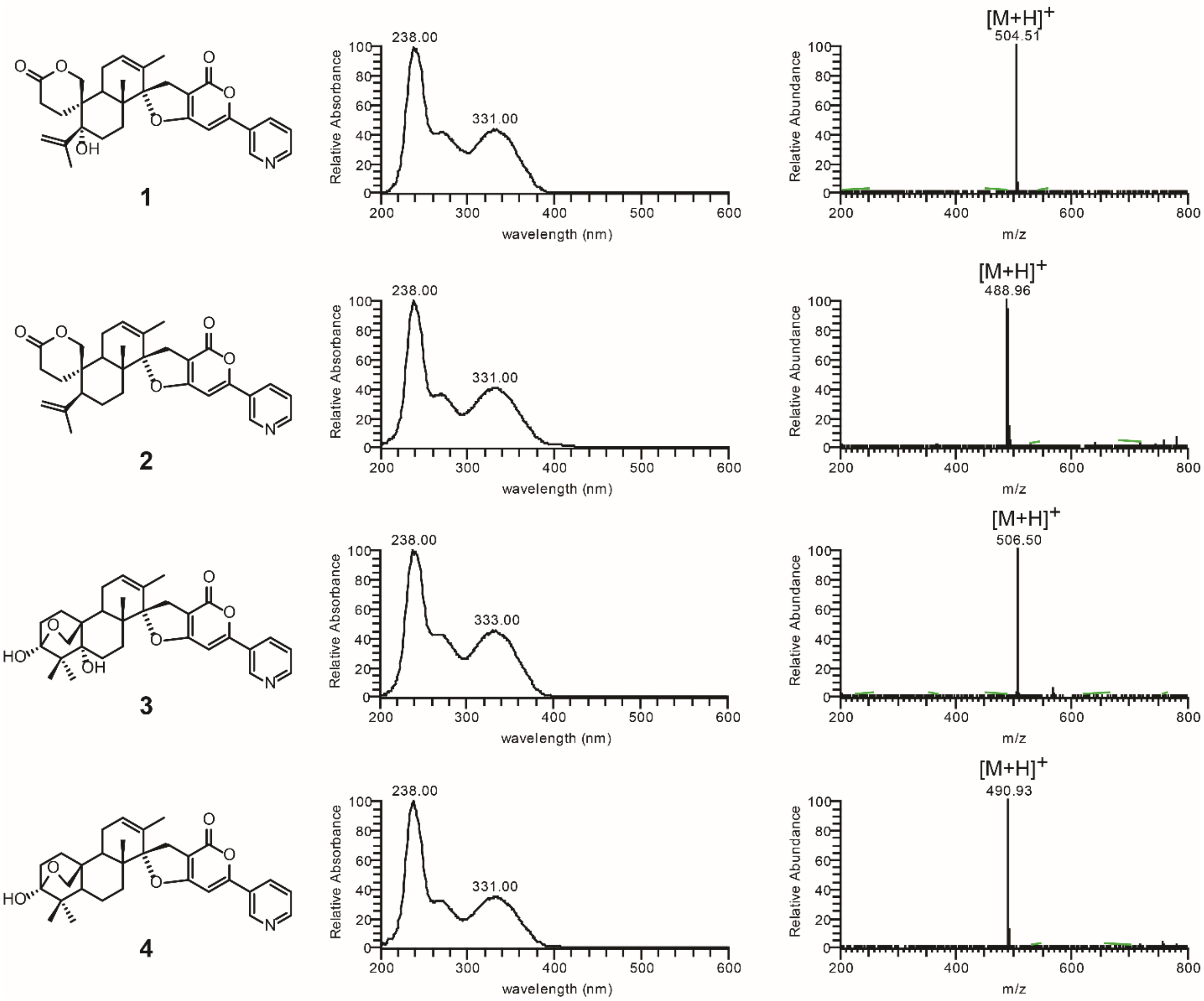

Figure S3. UV-Vis and ESI-MS spectra of compounds isolated from this study.

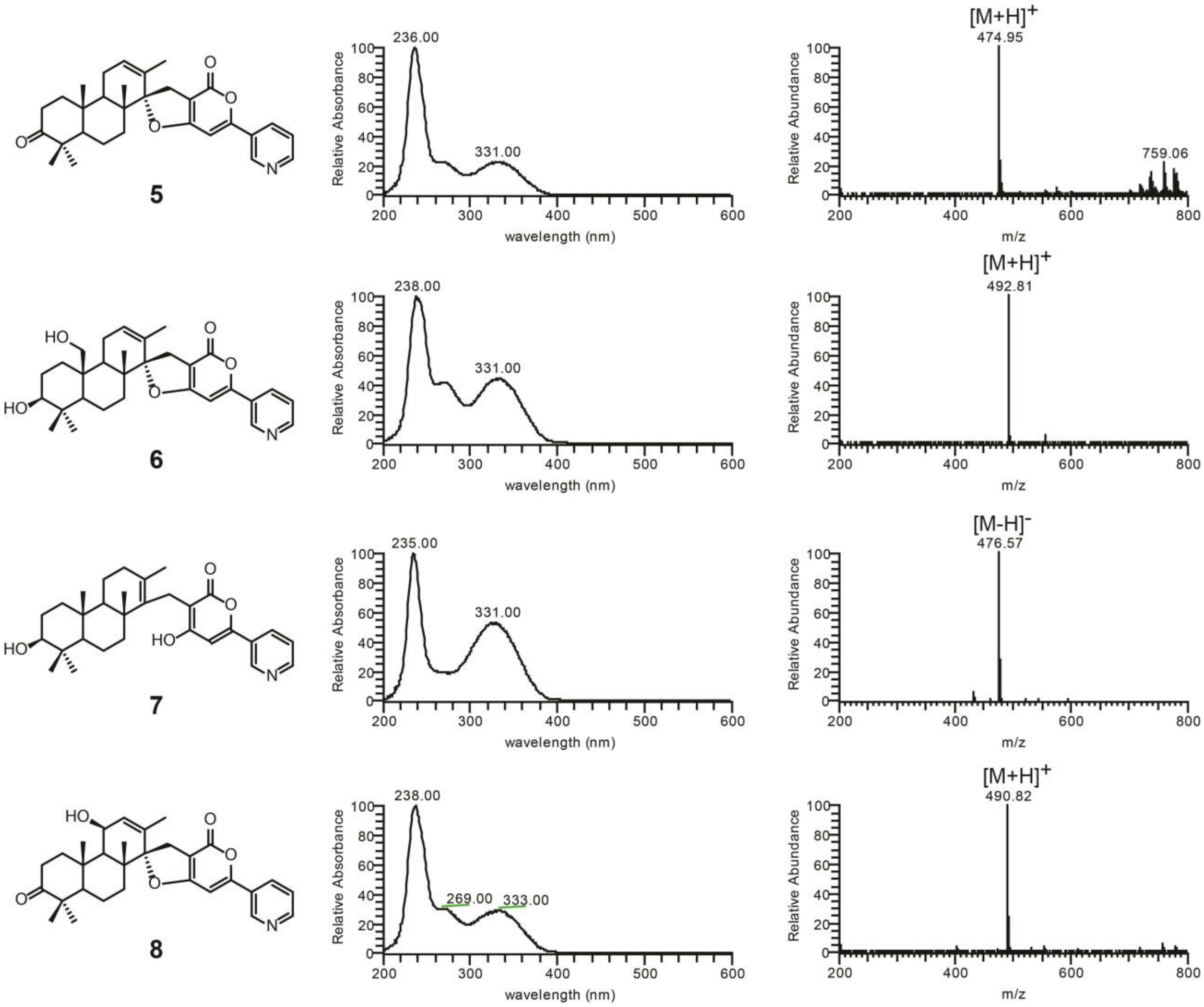

Figure S3 (continued). UV-Vis and ESI-MS spectra of compounds isolated from this study.

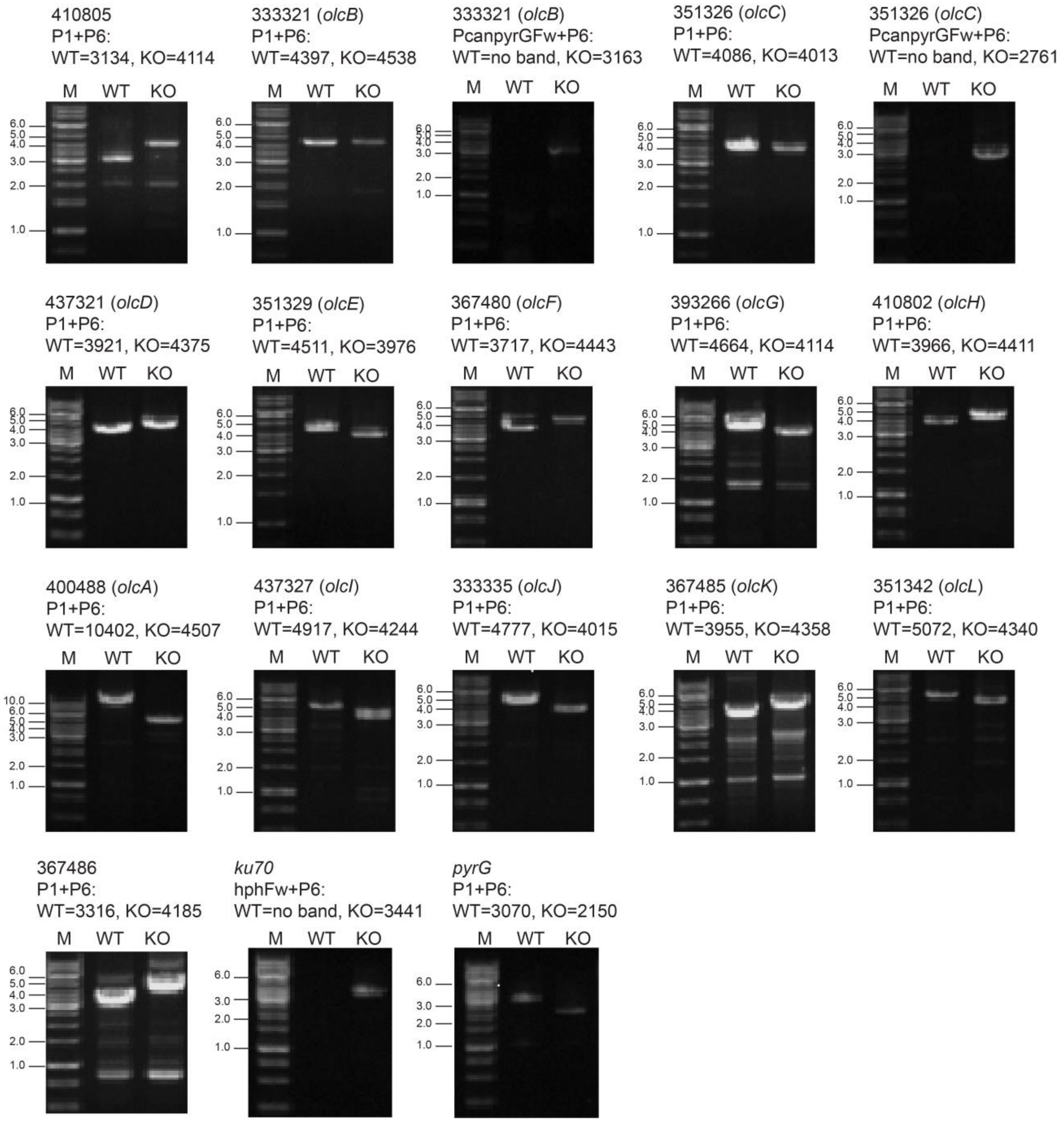

Figure S4. Results of diagnostic PCR for deletion strains.

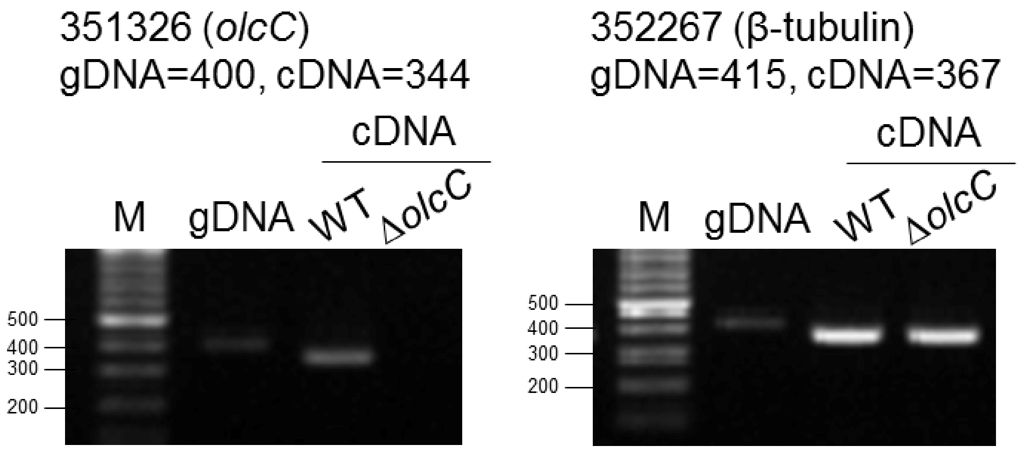

Figure S5. Result of RT-PCR analysis for *olcC*.

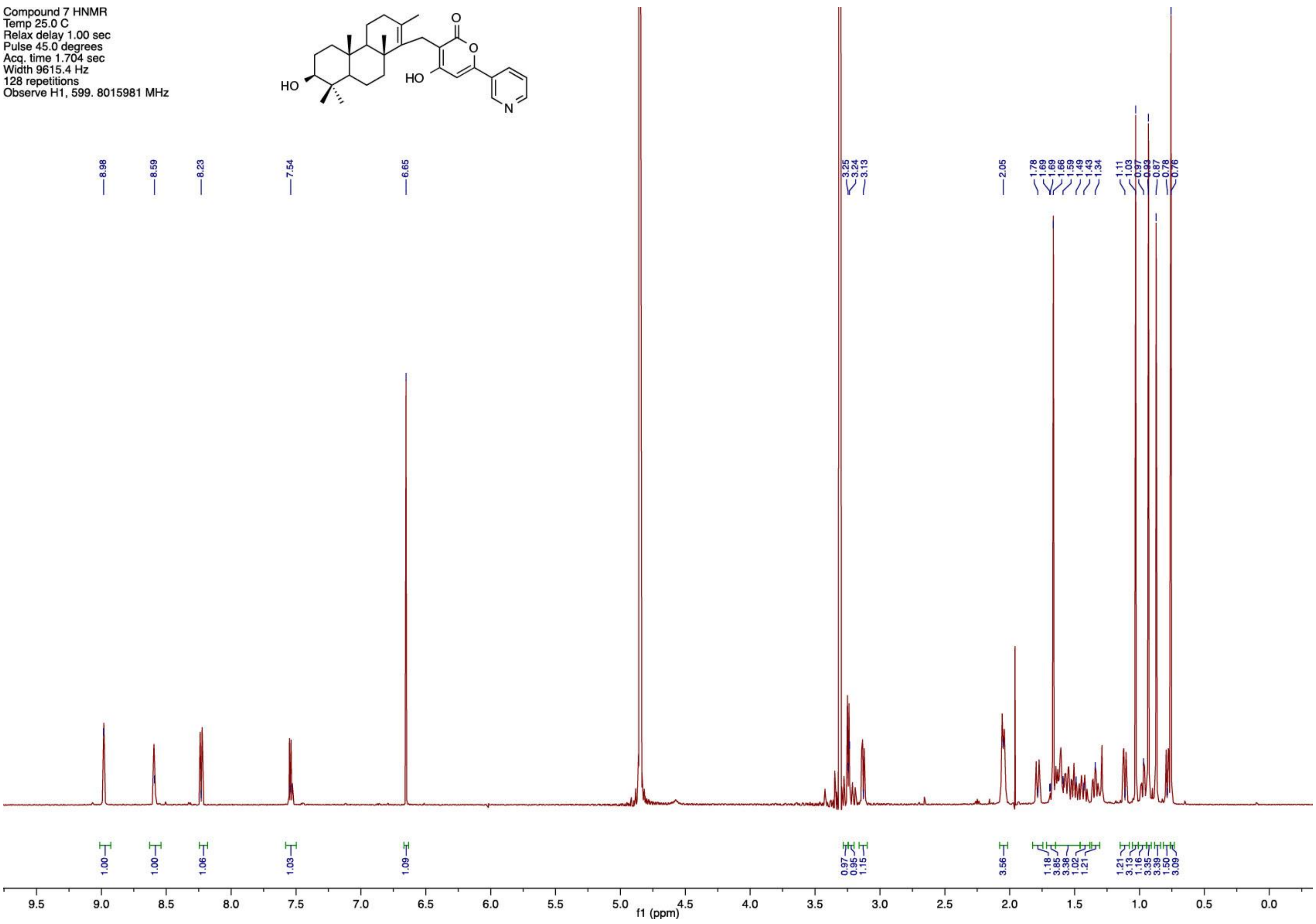

Figure S6. ^{1}H-NMR spectrum of compound **7** in CD_3OD

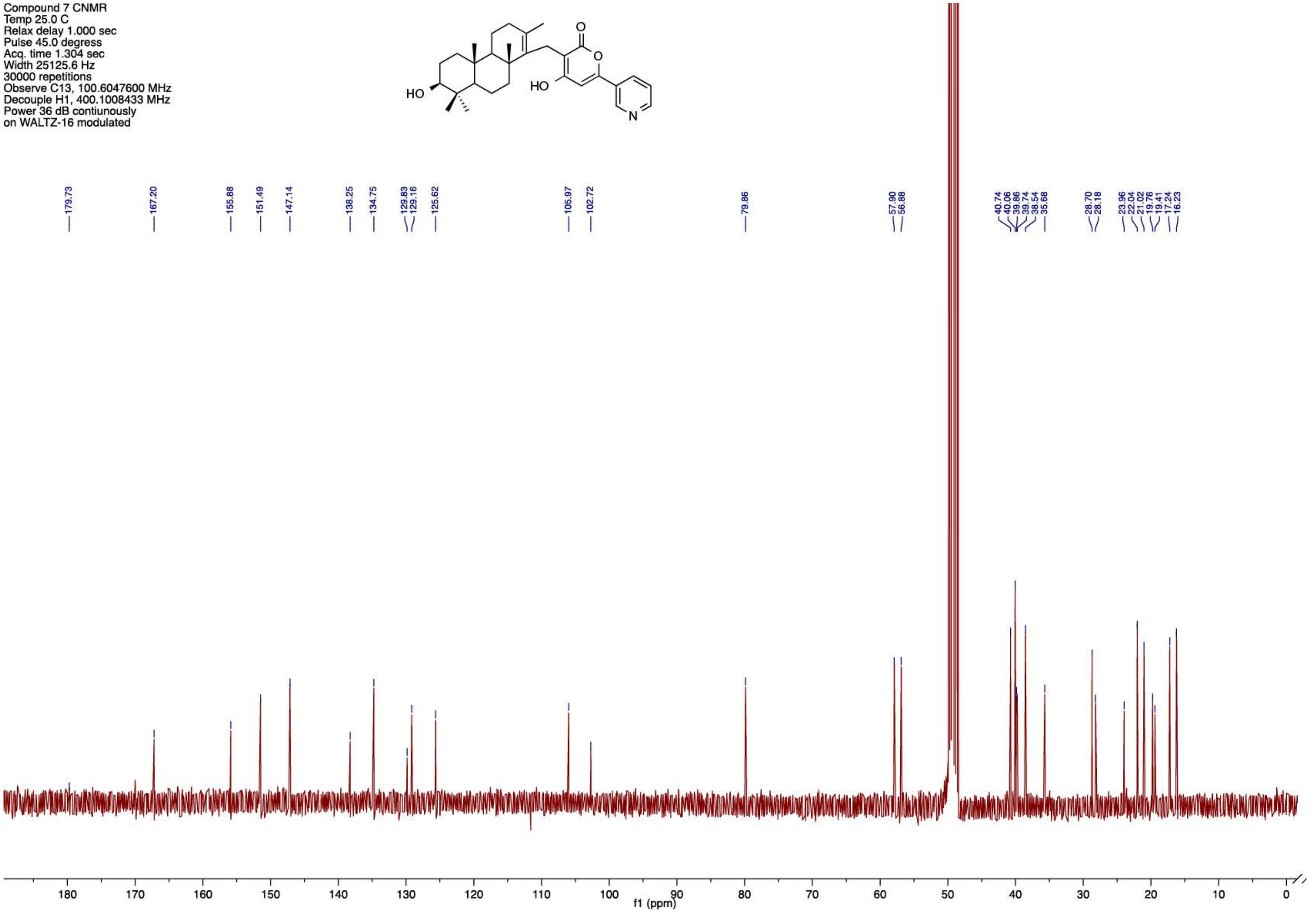

Figure S7. ^{13}C-NMR spectrum of compound **7** CD_3OD

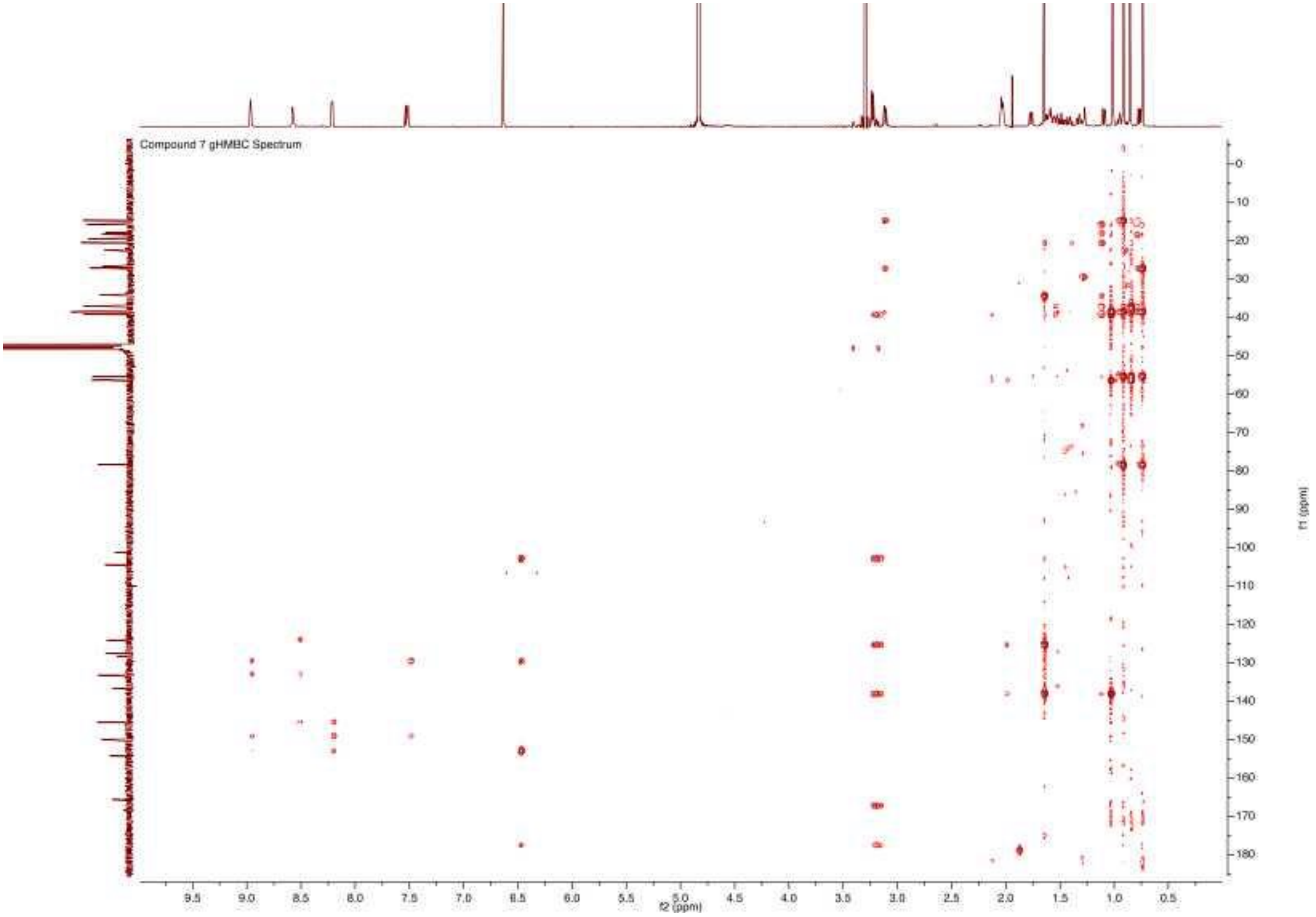

Figure S8. ^{1}H-^{13}C gHMBC spectrum of compound 7 in CD_3OD

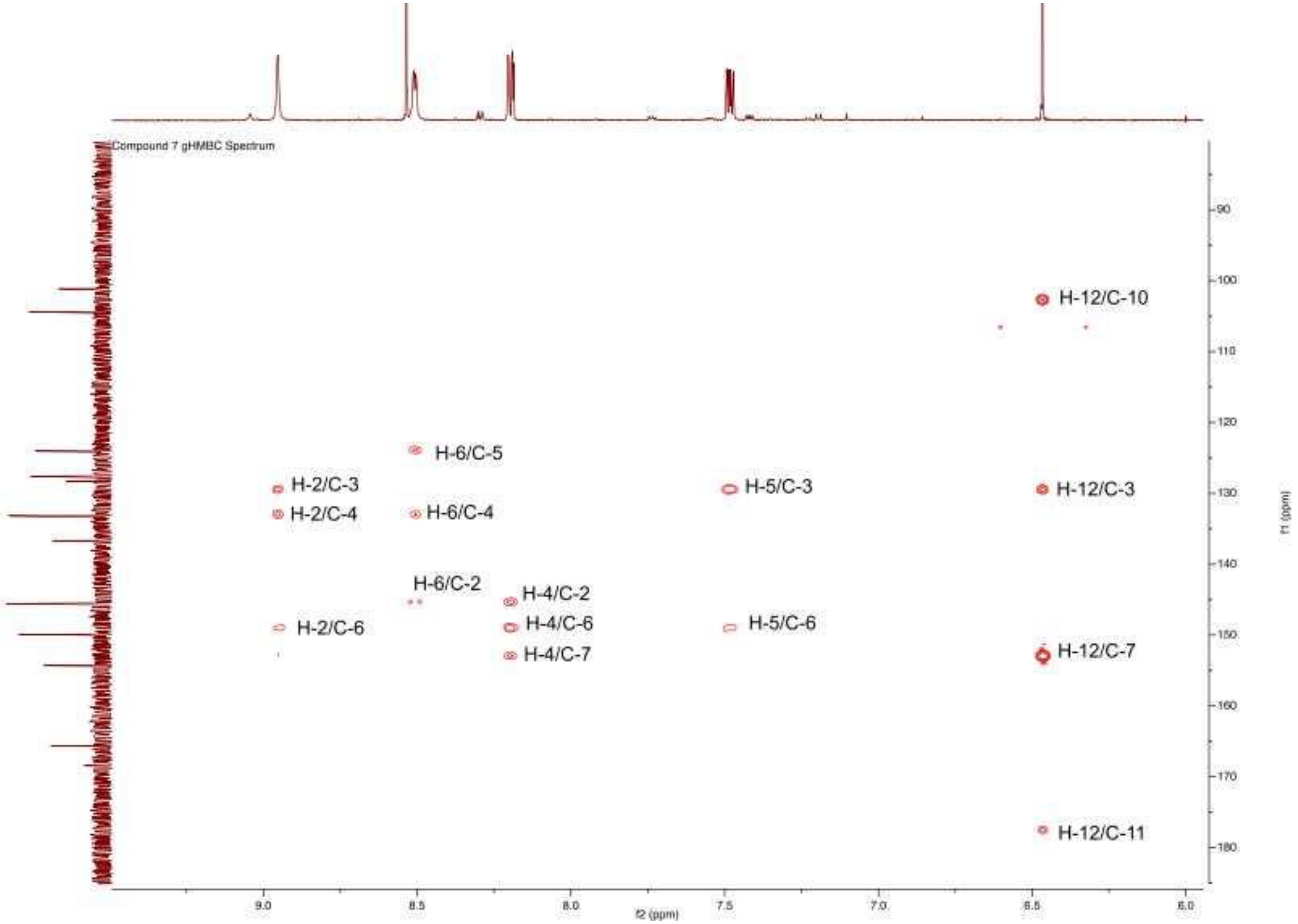

Figure S9. ^{1}H-^{13}C gHMBC spectrum of compound 7 in CD_3OD with labeled signals (Part 1/3)

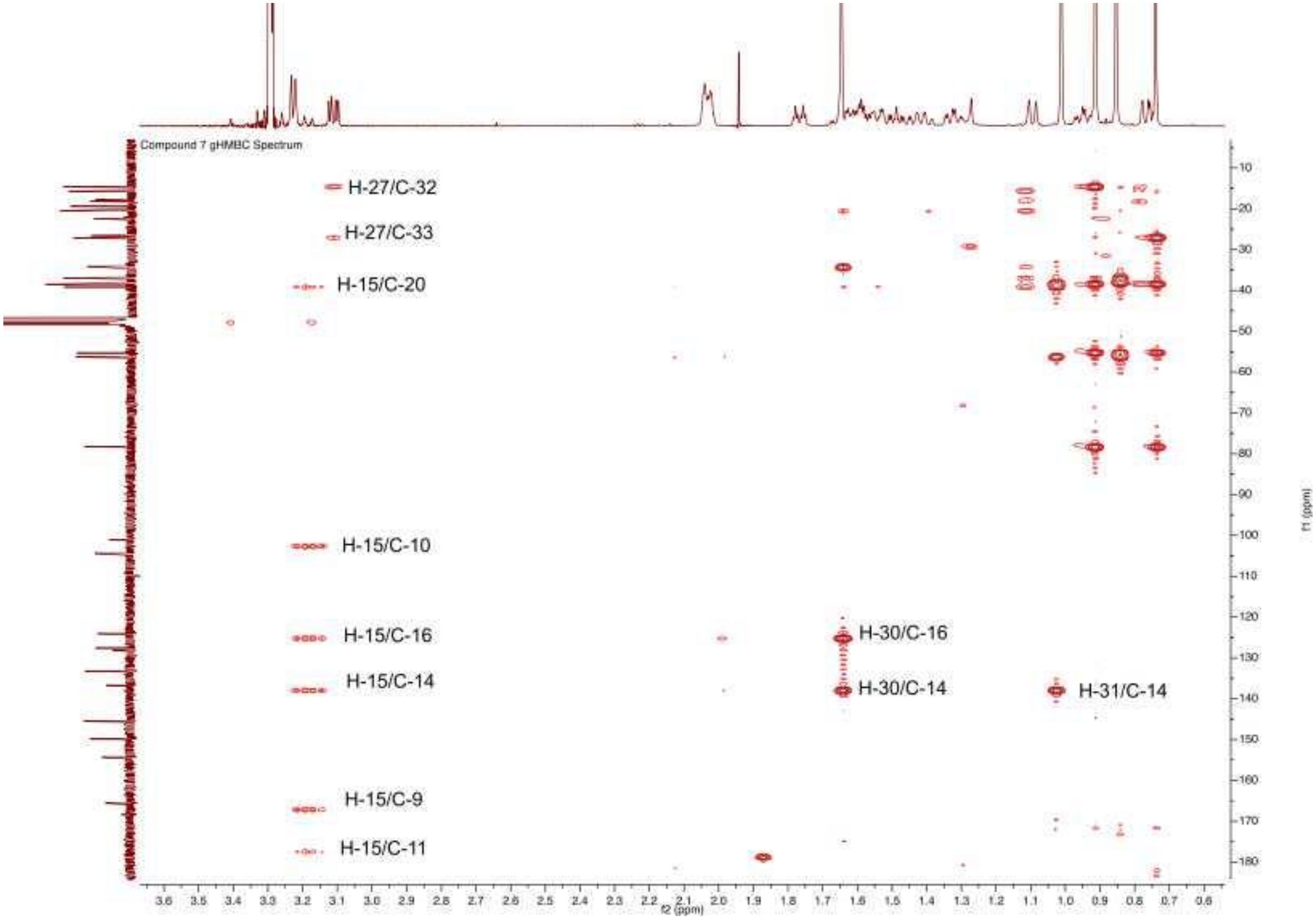

Figure S10. ^{1}H-^{13}C gHMBC spectrum of compound 7 in CD_3OD with labeled signals (Part 2/3)

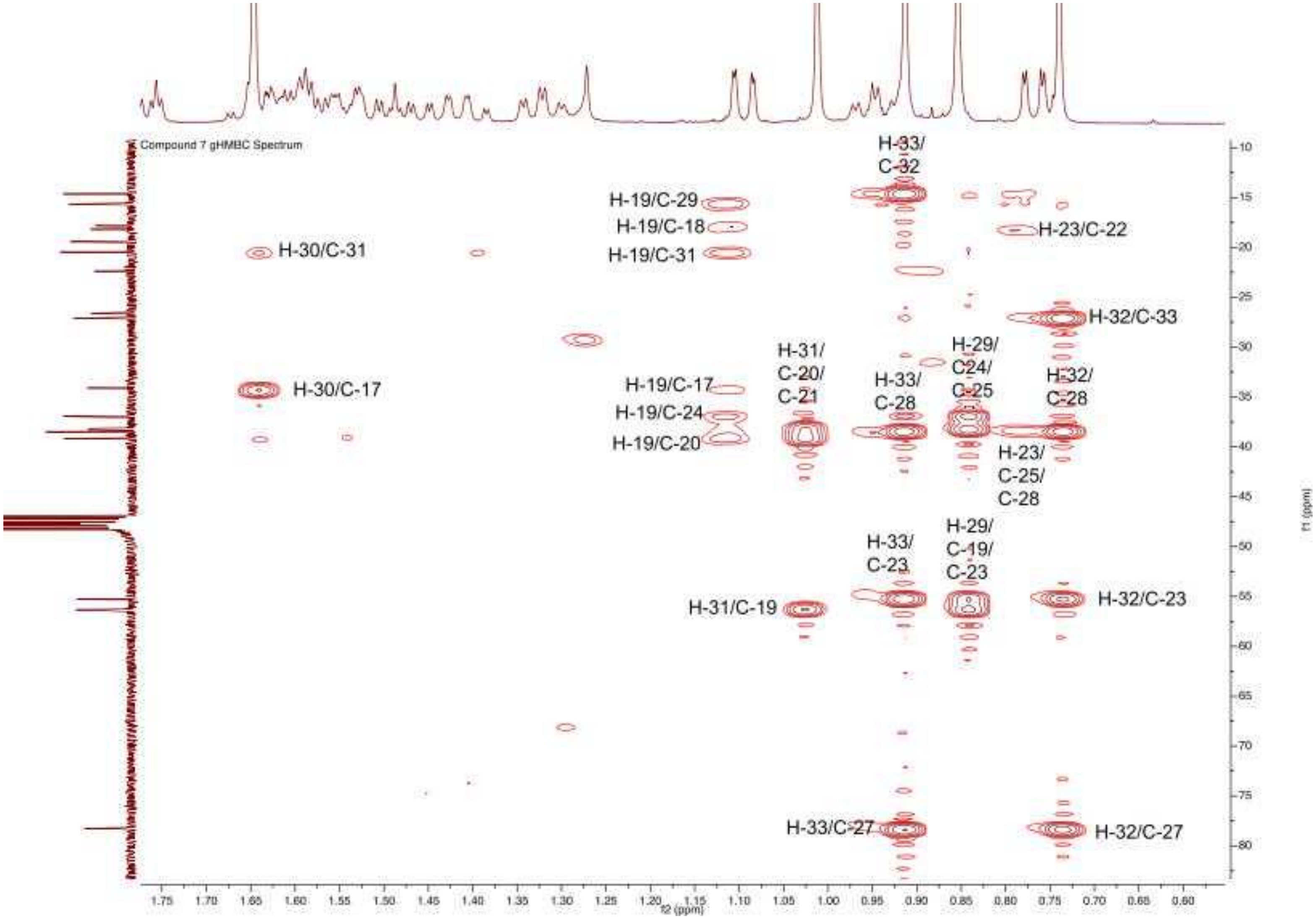

Figure S11. ^{1}H-^{13}C gHMBC spectrum of compound 7 in CD_3OD with labeled signals (Part 3/3)

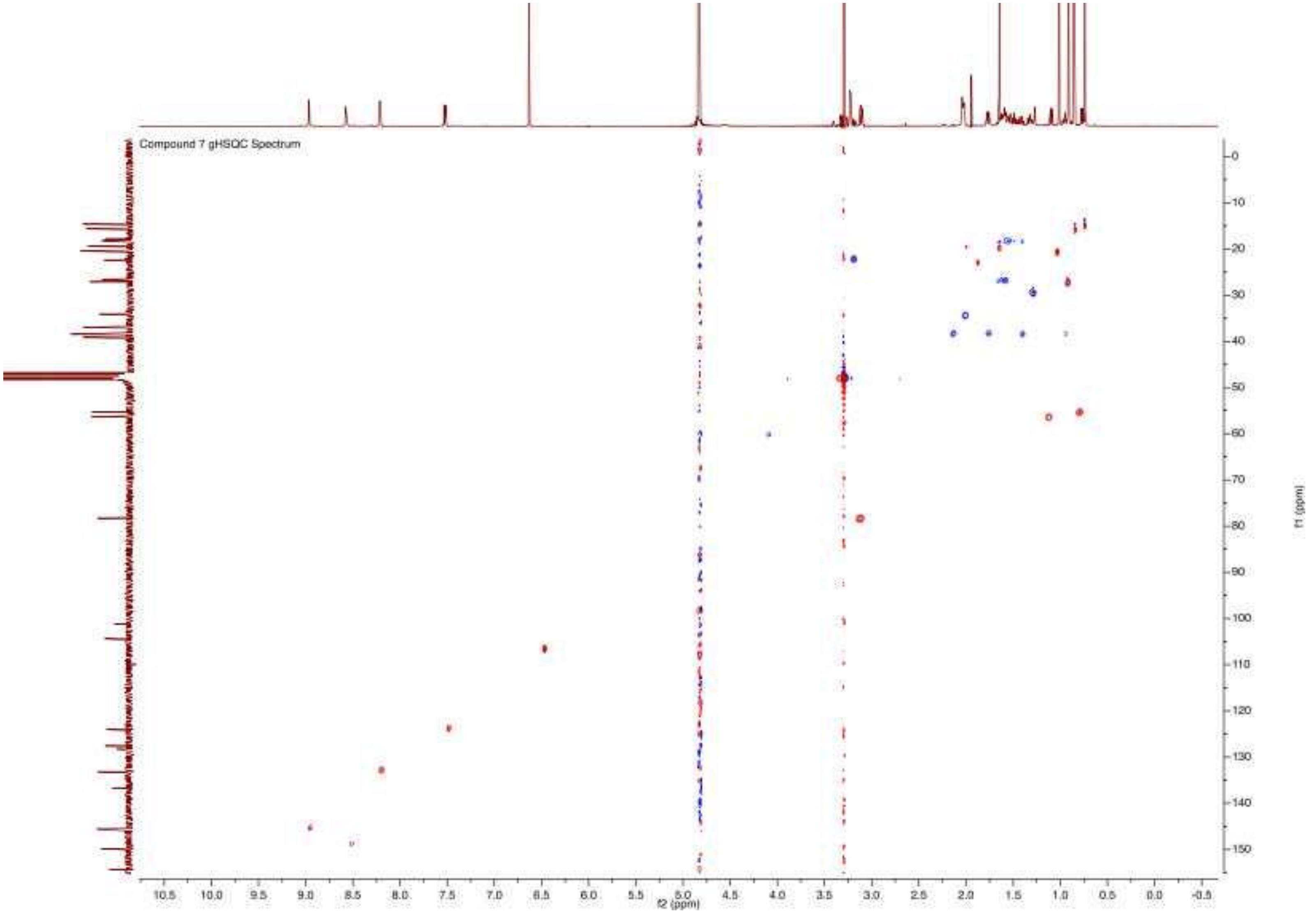

Figure S12. ^{1}H-^{13}C gHSQC spectrum of compound 7 in CD_3OD

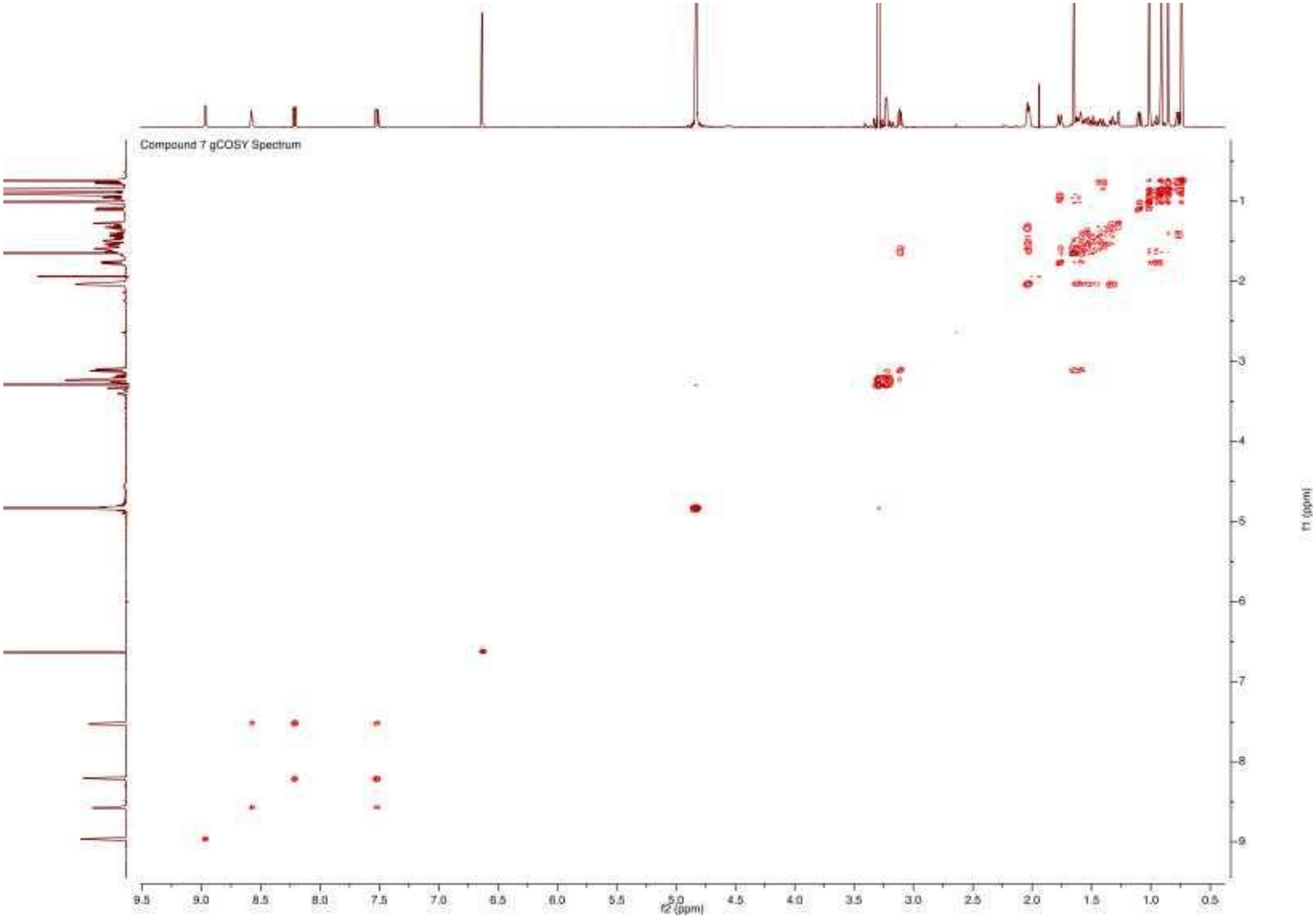

Figure S13. ¹H-¹H gCOSY spectrum of compound 7 in CD_3OD

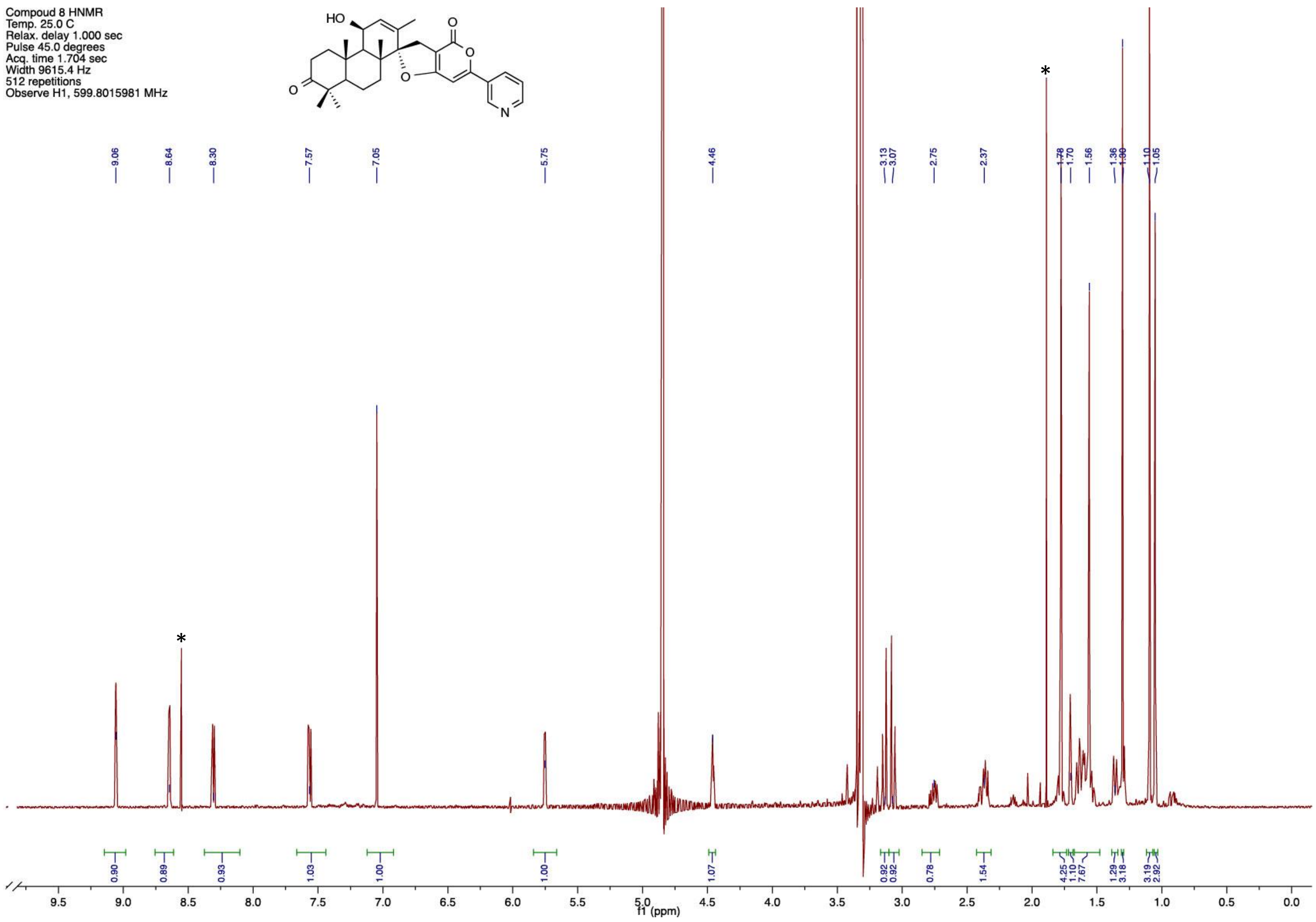

Figure S14. ^{1}H-NMR spectrum of compound **8** in CD_3OD**.** Peaks labeled with * were determined to be impurities based on 2D NMR spectral data which showed that they do not correlate with any other part of **8**. Both signals were also originally present in **7** and we were able to remove them by HPLC with 0.05 % of TFA in mobile phase. However, purification with TFA resulted in degradation of the compound **8**.

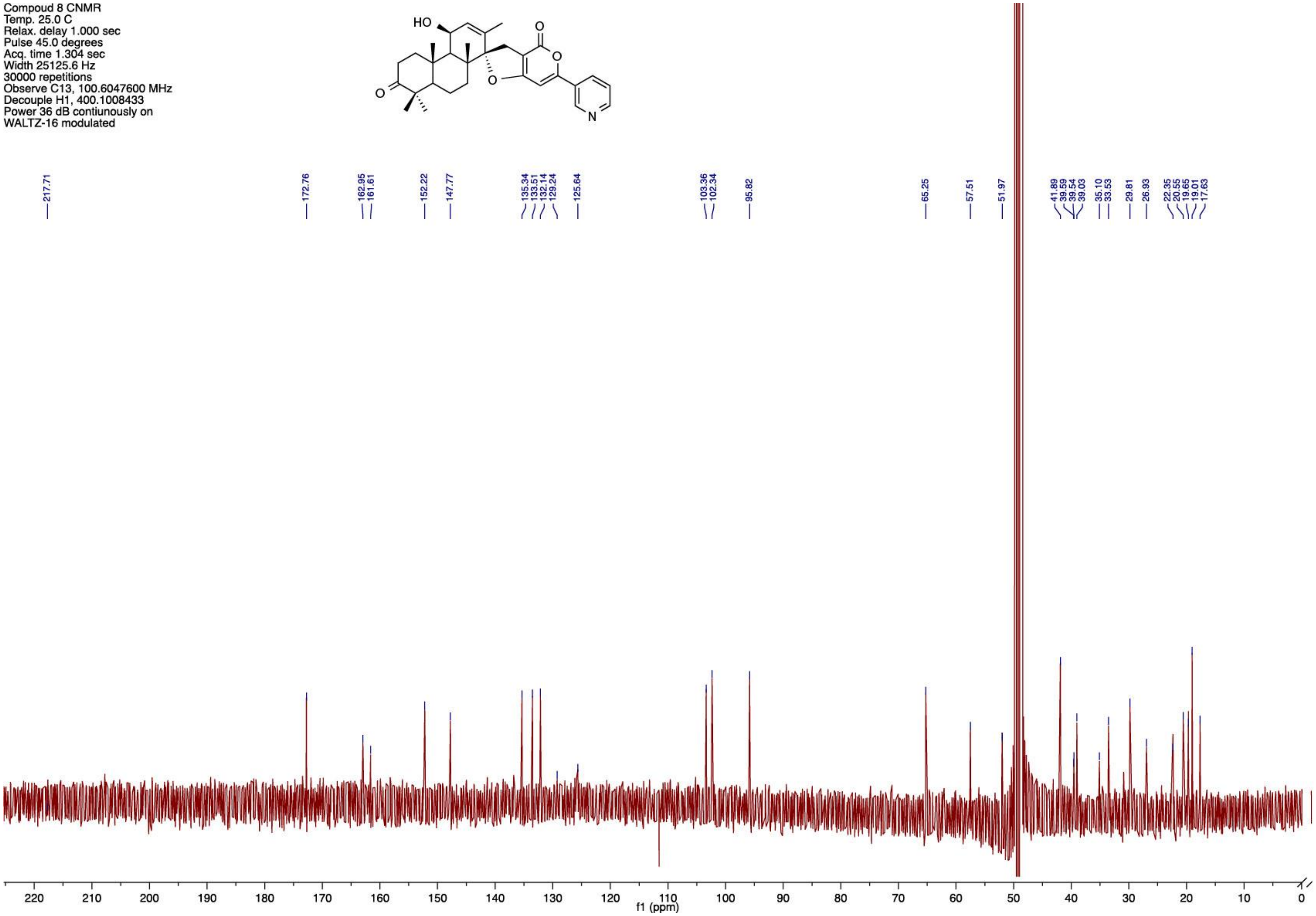

Figure S15. ^{13}C-NMR spectrum of compound **8** in CD_3OD

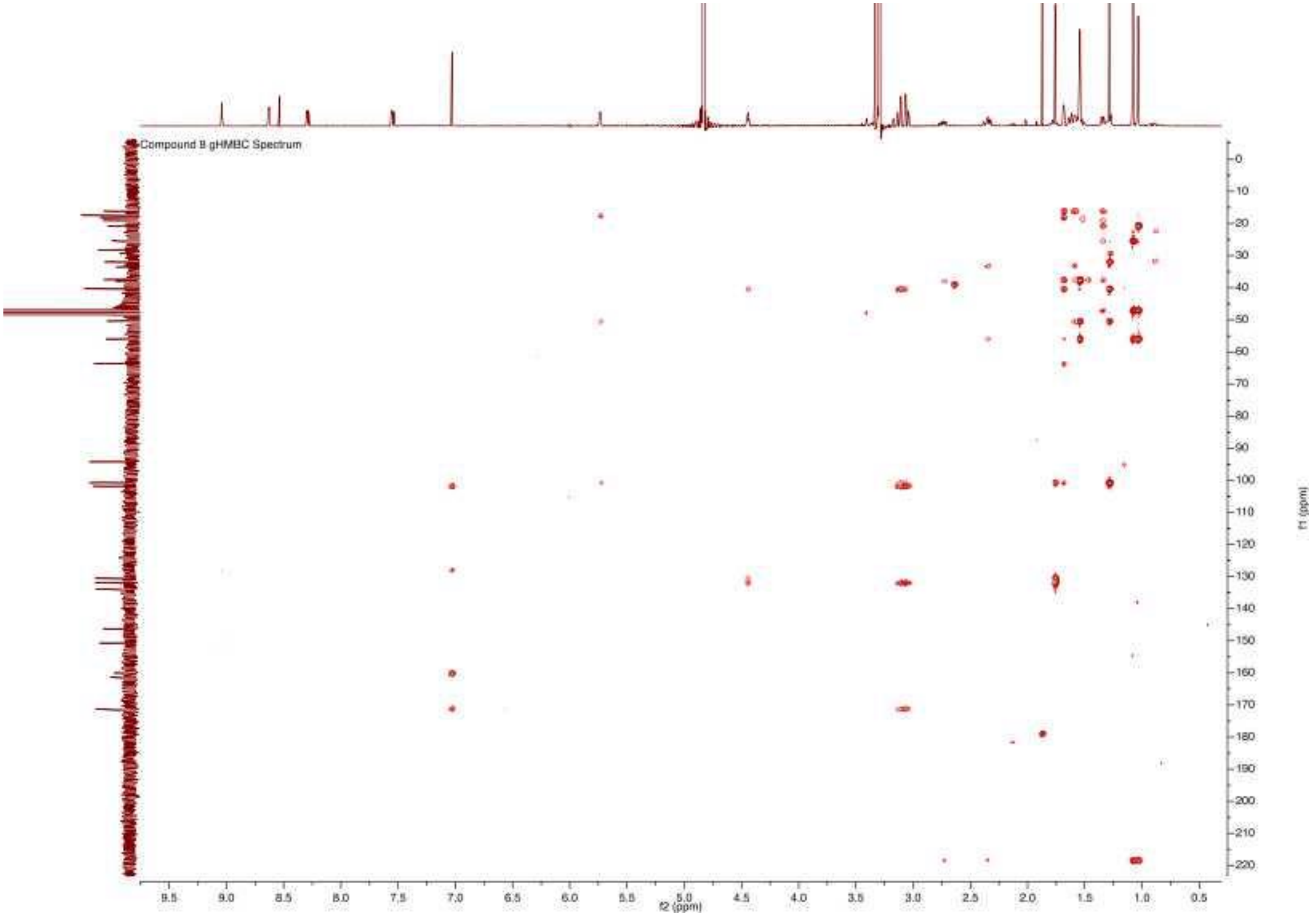

Figure S16. ^{1}H-^{13}C gHMBC spectrum of compound 8 in CD_3OD

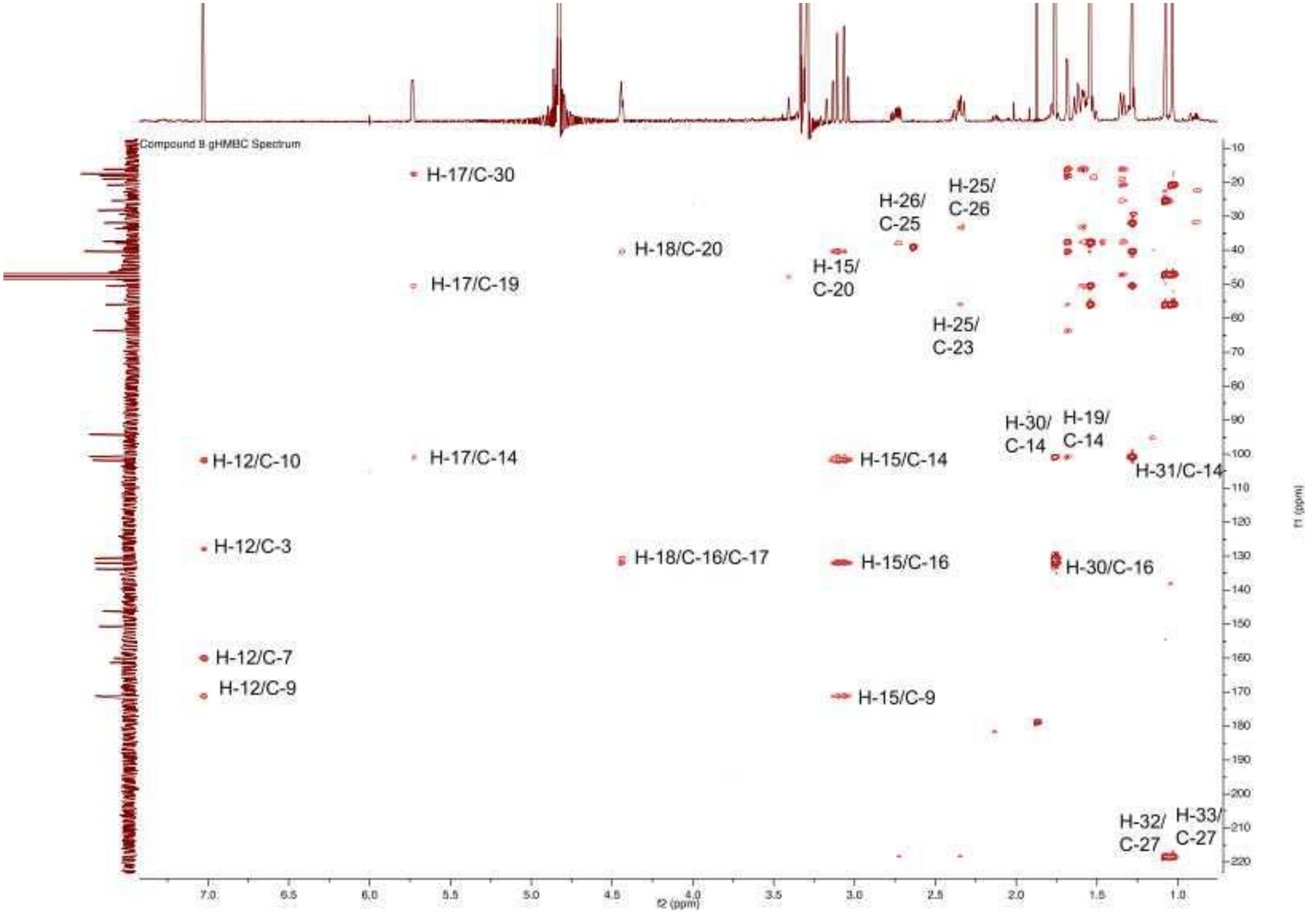

Figure S17. ^{1}H-^{13}C gHMBC spectrum of compound 8 in CD_3OD with labeled signals (Part 1/2)

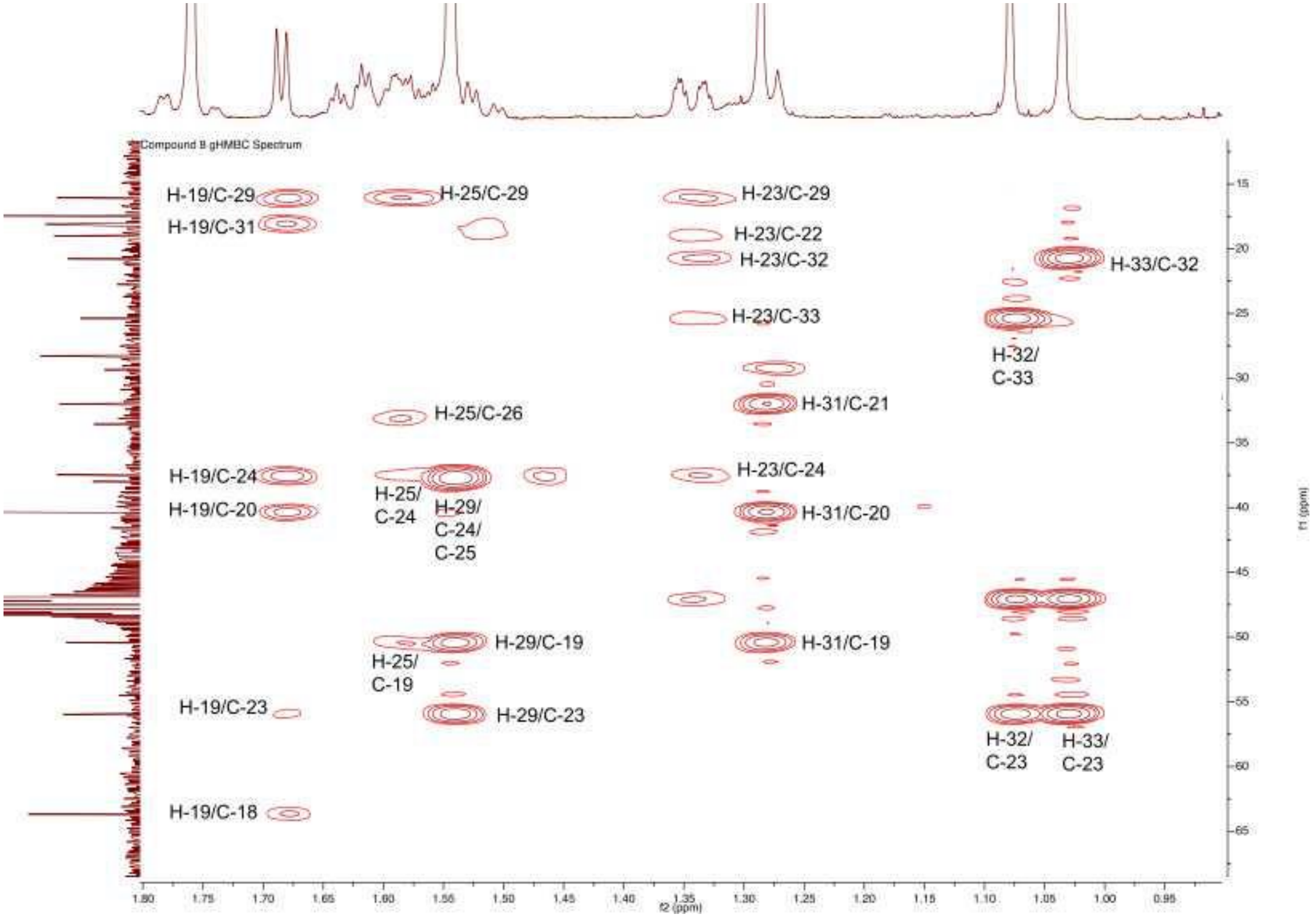

Figure S18. ^{1}H-^{13}C gHMBC spectrum of compound 8 in CD_3OD with labeled signals (Part 2/2)

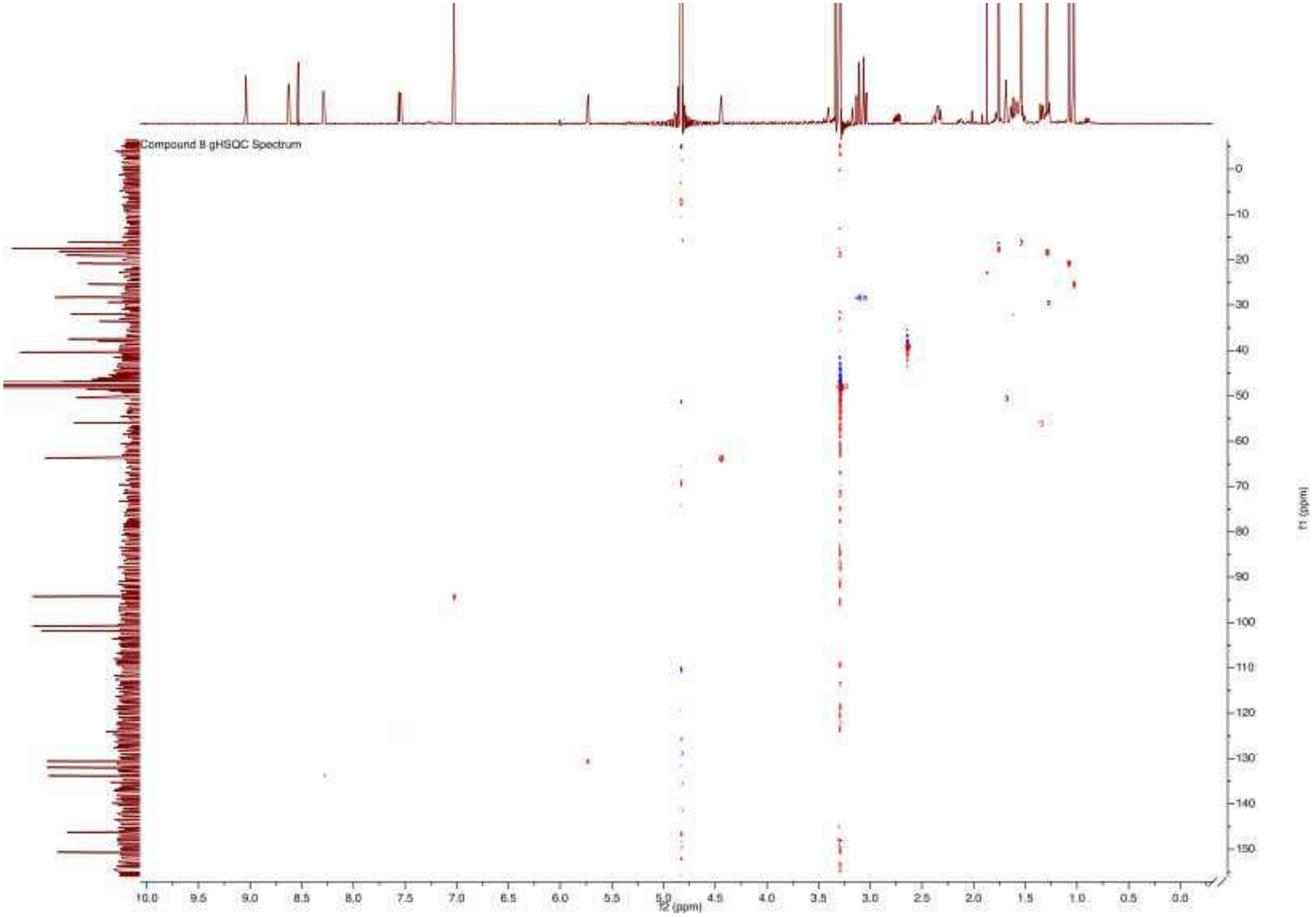

Figure S19. ^{1}H-^{13}C gHSQC spectrum of compound 8 in CD_3OD

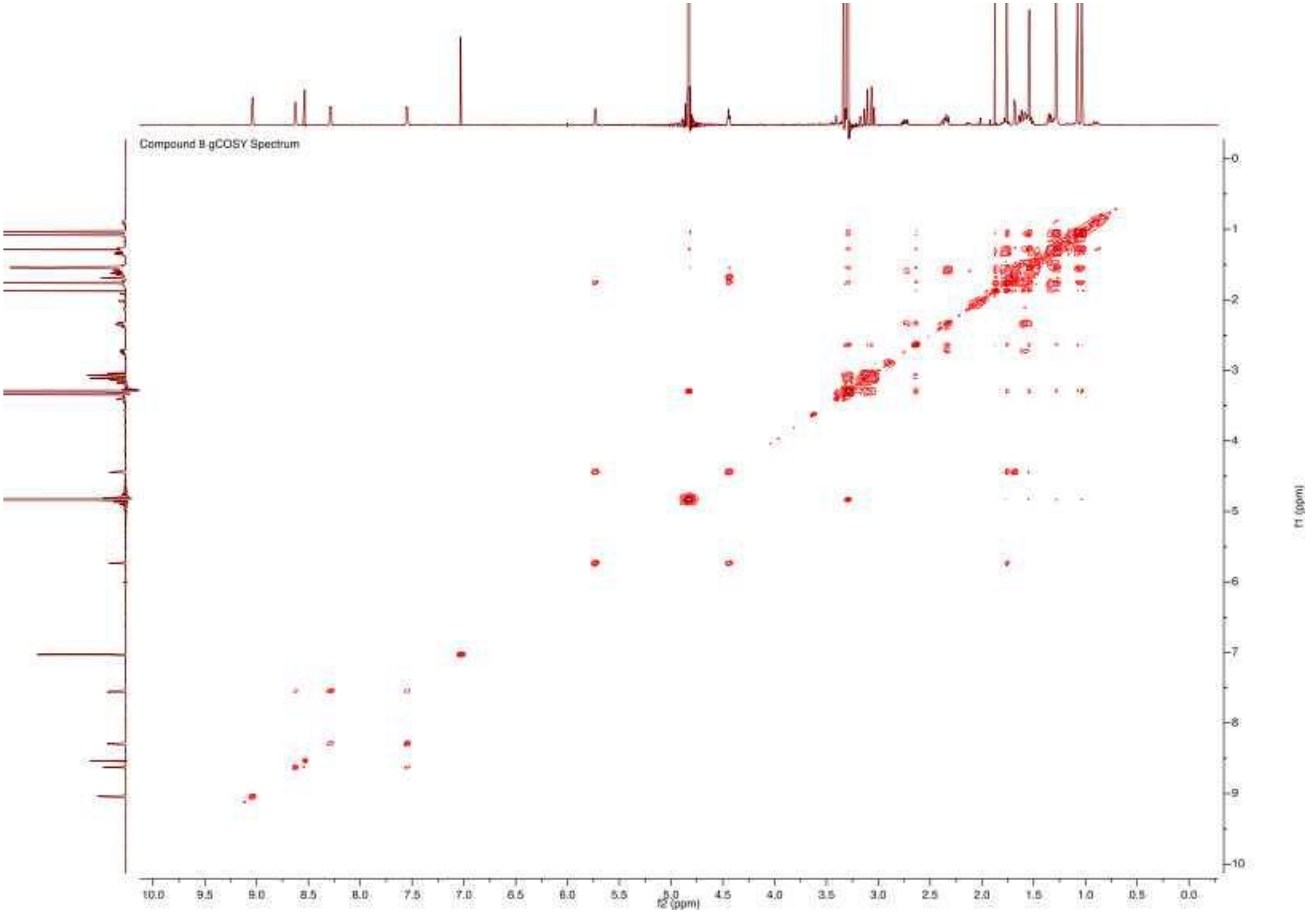

Figure S20. ^{1}H-^{1}H gCOSY spectrum of compound 8 in CD_3OD
